# Hunting monolignol transporters: membrane proteomics and biochemical transport assays with membrane vesicles of Norway spruce

**DOI:** 10.1093/jxb/eraa368

**Published:** 2020-08-10

**Authors:** Enni Väisänen, Junko Takahashi, Ogonna Obudulu, Joakim Bygdell, Pirkko Karhunen, Olga Blokhina, Teresa Laitinen, Teemu H Teeri, Gunnar Wingsle, Kurt V Fagerstedt, Anna Kärkönen

**Affiliations:** 1 Viikki Plant Science Centre, Organismal and Evolutionary Biology Research Programme, Faculty of Biological and Environmental Sciences, University of Helsinki, Helsinki, Finland; 2 Viikki Plant Science Centre, Department of Agricultural Sciences, University of Helsinki, Helsinki, Finland; 3 Department of Forest Genetics and Plant Physiology, Umeå Plant Science Centre (UPSC), Swedish University of Agricultural Sciences, Umeå, Sweden; 4 Department of Chemistry, Computational Life Science Cluster (CLiC), Umeå University, Umeå, Sweden; 5 Department of Chemistry, University of Helsinki, Helsinki, Finland; 6 Natural Resources Institute Finland (Luke), Production Systems, Plant Genetics, Helsinki, Finland; 7 Department of Microbiology and Immunology, Institute of Biomedicine, University of Gothenburg, Gothenburg, Sweden; 8 Chinese Academy of Sciences, China

**Keywords:** Lignin biosynthesis, monolignol transport, plasma membrane, proteomics, transporter proteins

## Abstract

Both the mechanisms of monolignol transport and the transported form of monolignols in developing xylem of trees are unknown. We tested the hypothesis of an active, plasma membrane-localized transport of monolignol monomers, dimers, and/or glucosidic forms with membrane vesicles prepared from developing xylem and lignin-forming tissue-cultured cells of Norway spruce (*Picea abies* L. Karst.), as well as from control materials, comprising non-lignifying Norway spruce phloem and tobacco (*Nicotiana tabacum* L.) BY-2 cells. Xylem and BY-2 vesicles transported both coniferin and *p*-coumaryl alcohol glucoside, but inhibitor assays suggested that this transport was through the tonoplast. Membrane vesicles prepared from lignin-forming spruce cells showed coniferin transport, but the *K*_m_ value for coniferin was much higher than those of xylem and BY-2 cells. Liquid chromatography-mass spectrometry analysis of membrane proteins isolated from spruce developing xylem, phloem, and lignin-forming cultured cells revealed multiple transporters. These were compared with a transporter gene set obtained by a correlation analysis with a selected set of spruce monolignol biosynthesis genes. Biochemical membrane vesicle assays showed no support for ABC-transporter-mediated monolignol transport but point to a role for secondary active transporters (such as MFS or MATE transporters). In contrast, proteomic and co-expression analyses suggested a role for ABC transporters and MFS transporters.

## Teaser

Transport of monolignols into the apoplastic space is a major event in woody stems. This work identifies candidate transporters in Norway spruce through large-scale LC-MS/MS membrane proteomics and gene co-expression analyses.

## Introduction

Lignification of plant cell walls is a developmental process in water-transporting xylem cells and support-giving sclerenchyma cells. In addition, it can be an induced as a protective response against stresses. In lignification, hydroxycinnamic alcohols (monolignols) form a polymer in the cell wall. The biosynthetic process of lignification includes the production of monolignols through the monolignol biosynthetic pathway, a branch of the phenylpropanoid pathway. Next, monolignols are secreted into the cell wall, oxidized to radicals, and the radicals are polymerized into lignin, a complex network of linkages between monolignols (Perkins *et al.*, 2019). The process is mediated by a wide variety of enzymes in multiple locations.

The plasma membrane (PM) serves the plant cell as a controlling boundary between the cell wall and the cytoplasm. This boundary has many roles, including maintaining cellular ionic homeostasis, creating concentration gradients, and thus regulating biochemical reactions, and detecting and mediating signals between the apoplast and the symplast. In addition, the PM is important for cell wall biosynthesis because cellulose is produced directly at the PM. The biosynthesis of lignin is separated in space: monolignol biosynthesis takes place in the cytoplasm and lignin polymerization occurs in the cell wall. Thus, monolignols require transportation through the PM to the site of polymerization. According to biochemical data, transport of monolignols through the PM involves enzymatic transporters (Miao and Liu, 2010; Alejandro *et al.*, 2012). Still, passive diffusion has a likely role in aglycone transport (Boija and Johansson, 2006; Vermaas *et al.*, 2019), whereas Golgi-mediated transport does not seem probable (Kaneda *et al.*, 2008).

The transport mechanism of monolignols has remained as a mystery (Perkins *et al.*, 2019). Even the transported form is not yet known. The most favored candidates, monolignol glucosides (such as coniferyl alcohol glucoside, or coniferin), are non-toxic, non-reactive, and readily detectable molecules in the developing secondary xylem of many gymnosperms (Morikawa *et al.*, 2010; Terashima *et al.*, 2016) and some angiosperms (e.g. *Magnolia*; Tsuji *et al.*, 2004). Coniferin β-glucosidases, which have been detected in, for example, developing xylem of lodgepole pine (*Pinus contorta*; Dharmawardhana *et al.*, 1995), cleave the sugar moiety, leaving the free alcohol form ready to react with peroxidases/laccases and the growing lignin polymer. Results using coniferyl alcohol and coniferin in assays with membrane vesicles prepared from aerial tissues of *Arabidopsis thaliana* (Miao and Liu, 2010) suggest a role for PM-located ABC transporter(s) in coniferyl alcohol transport. In addition, Alejandro *et al.* (2012) proposed that a G-type ABC transporter, AtABCG29, functions in *p*-coumaryl alcohol transport at the PM in Arabidopsis root endodermis and in vascular tissues. In contrast, several tree species show H^+^-antiporter-dependent transport of coniferin through the tonoplast and endomembranes (Tsuyama *et al.*, 2013). Recently, *p*-coumaryl alcohol glucoside was shown to be transported via the same mechanism in hybrid poplar (*Populus sieboldii* × *P. grandidentata*) and Japanese cypress (*Chamaecyparis obtusa*), supporting the role of vesicular transport in monolignol transport (Tsuyama *et al.*, 2019).

In addition, transport of dilignols seems possible. The presence of conjugates of sinapyl alcohol and *p*-coumaric acid in grass lignin suggests that there is a system that transports dimers into the apoplast, since a transferase making these conjugates exists in the cytoplasm (Hatfield et al., 2008, 2009). It seems possible that the first step of polymerization, dimerization, occurs partly in the cytosol, and the dimer is transported to the cell wall and incorporated into the growing polymer.

In the current study, membrane proteomic analysis of developing xylem with active lignification and lignin-forming tissue-cultured cells (Kärkönen *et al.*, 2002) of Norway spruce (*Picea abies*) was conducted. As a comparison, the membrane proteome of developing phloem, which does not lignify (except stone cells), was investigated. Microsomal vesicles (microsomal fraction; MF) are a heterogeneous mixture of cellular membranes. Partially enriched PM vesicles (upper phase fraction; UP) (Kärkönen *et al.*, 2014) were prepared from MF material and their proteomes were analyzed by liquid chromatography-mass spectrometry (LC-MS/MS). To take a closer look at membrane-localized, putatively lignification-related proteins, protein identifications were compared with several published gene expression studies (Nystedt *et al.*, 2013; Jokipii-Lukkari *et al.*, 2017; Laitinen *et al.*, 2017; Blokhina *et al.*, 2019), and candidates for monolignol transport and other lignification-related processes were identified. Biochemical monolignol transport assays with multiple candidate transport forms showed that MF prepared from developing xylem, but not those from developing phloem, had a H^+^-gradient-dependent, secondary active, tonoplast-localized transport of coniferin and *p*-coumaryl alcohol glucoside. A strikingly similar, active tonoplast-localized coniferin and *p*-coumaryl alcohol glucoside transport was detected in MF prepared from non-lignifying control material, consisting of tissue-cultured Bright Yellow-2 (BY-2) cells of tobacco (*Nicotiana tabacum*). This observation questions whether this transporter is dedicated to lignin biosynthesis. In addition, MF isolated from the lignin-forming cultured cells had a transporter able to transport coniferin, but its affinity for coniferin was much lower than those in xylem or BY-2 cells. In combination with the proteomic data, correlation analyses using existing Norway spruce RNA-seq data were utilized to identify candidate monolignol transporters.

## Materials and methods

### Plant material

In each of the years 2011, 2013, and 2014, an approximately 40-year-old Norway spruce (*Picea abies* [L.] Karst., clone E8504) tree, grown in Ruotsinkylä, southern Finland, was felled in late June, when xylem lignification was actively going on. Developing xylem and phloem were collected as described in Väisänen *et al.* (2018).

A Norway spruce callus culture (line A3/85) that forms extracellular lignin (Simola *et al.*, 1992) was maintained on a solid medium according to Kärkönen *et al.* (2002). Cells were transferred into a liquid medium (Simola and Santanen, 1990) ~2.5 weeks after subculturing. The cells were cultivated in the liquid culture for ~5 days, then collected, washed with water, and stored at –80°C (Kärkönen *et al.*, 2014).

Tobacco (*Nicotiana tabacum* L.) Bright Yellow-2 (BY-2) cells (Nagata *et al.*, 1992) were cultured in a modified Murashige and Skoog (1962) medium, pH 5.8, supplemented with 0.9 μM 2,4-dichlorophenoxyacetic acid, 3 % (w/v) sucrose, 3 μM thiamine HCl, and 2.7 mM KH_2_PO_4_. The cells were grown in the dark, in 50 ml aliquots in 300 ml Erlenmeyer flasks on a rotary shaker (100 rpm) at 25 °C, and subcultured weekly. Seven days after subculturing, the cells were collected, washed, and stored at –80°C.

### Membrane preparations

Spruce xylem and phloem, lignin-forming cultured cells, and BY-2 cells were ground as described in Väisänen *et al.* (2018). Out of the ground material, microsomal vesicles (MF) were prepared. Aqueous polymer two-phase partitioning (Larsson *et al.*, 1994) as optimized for developing spruce xylem and cultured cells (Väisänen *et al.*, 2018) was used to prepare enriched PM vesicles (UP). MF was prepared from the BY-2 cells using the same procedure as for xylem. Membrane fractions from each material were stored at –80 °C in storage buffer [10 mM MOPS-KOH, pH 7.5, 0.33 M sucrose and 1 mM dl-dithiothreitol (DTT)] until use.

For proteomic analyses, the UP and MF fractions of spruce xylem, phloem, and tissue-cultured cells were washed with washing buffer [10 mM MOPS-KOH, pH 7.5, 150 mM KCl, 2 mM MgCl_2_, 1 mM EDTA, and 0.01% (w/v) Triton X-100] to reduce the amount of soluble proteins entrapped inside the membrane vesicles or weakly attached to the membranes. Each membrane sample was mixed with the washing buffer, incubated on ice for 30 min, and pelleted at 110 000 *g* for 45 min at 4 °C. The washed membrane pellet was resuspended in storage buffer, frozen in liquid nitrogen, and stored at –80 °C.

For determination of protein amounts, the membrane samples were diluted with storage buffer supplemented with 0.01% (w/v) Triton X-100. Proteins were measured with a Bio-Rad protein assay (based on Bradford, 1976) with bovine serum albumin (BSA) as a standard.

### Substrates tested in the transport assays

Coniferyl alcohol, *p*-coumaryl alcohol, *p*-coumaryl alcohol 4-*O*-glucoside, pinoresinol, pinoresinol diglucoside, lariciresinol, and isolariciresinol of analytical grade were purchased from Sigma-Aldrich. β-*O*-4 erol was a kind gift from Prof. Jussi Sipilä and Dr Paula Nousiainen (Department of Chemistry, University of Helsinki, Finland). The synthesis of ^14^C-coniferin is described in Supplementary Protocol S1 at *JXB* online.

**Fig. 1.**
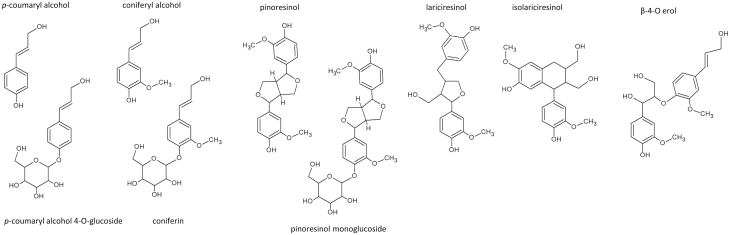
Chemical structures of compounds tested for transport.

Pinoresinol monoglucoside was prepared from pinoresinol diglucoside by enzymatic digestion with β-glucosidase (from almonds, Sigma; 0.72 U mg^–1^ pinoresinol diglucoside) in 50 mM MES buffer, pH 5.0, at 37 °C for 1 h. A preparative thin layer chromatography was run with acetone:ethylacetate:water (10:10:1), the spot between pinoresinol and pinoresinol diglucoside was scraped off, and the compound was extracted twice with 100% methanol and once with water. The identity of the compound as pinoresinol monoglucoside was confirmed by HPLC. ^14^C-coniferyl alcohol was prepared from ^14^C-coniferin by overnight enzymatic digestion at 36 °C immediately before each transport experiment. The reaction contained 170 kBq ml^−1 14^C-coniferin, 0.5 U ml^−1^ β-glucosidase (from almonds, Sigma), 50 mM Na acetate buffer, pH 5.0, and 0.9 mM non-radioactive coniferyl alcohol (added to the reaction as a protectant). A thin layer chromatography run followed by phosphoimaging analysis suggested that the reaction was complete. The final concentration of coniferyl alcohol (labelled+non-labelled) after hydrolysis was 5.7 mM. The chemical structures of the substrates are shown in Fig. 1.

### Transport assays

To study the membrane transport system(s) and the monolignol transport form(s), a vesicle assay was set up. Non-washed MF (10–200 µg, based on protein amount) were incubated at 25 °C in 100–200 µl of reaction buffer (50 mM MOPS-KOH, pH 7.5, 0.33 M sucrose, and 0.01% BSA) with 100 µM of substrate (^14^C-coniferin, ^14^C-coniferyl alcohol, non-labeled *p*-coumaryl alcohol, *p*-coumaryl alcohol glucoside, or the dimers pinoresinol, pinoresinol monoglucoside, lariciresinol, isolariciresinol, or β-*O*-4 erol) and with or without the addition of 5 mM Mg ATP, or 5 mM Na ATP supplemented with 8 mM MgCl_2_. The reaction was started with the addition of ATP (inhibitor experiments) or vesicles (all other experiments). The inhibitors used were 1 mM vanadate (Sigma-Aldrich; activated as described in Gordon, 1991), 50 µM gramicidin (Sigma-Aldrich; a mixture of gramicidins A, B, C, and D), 20 µM carbonyl cyanide *m*-chlorophenyl hydrazone (CCCP) (Sigma-Aldrich), or 1 µM bafilomycin A1 (Sigma-Aldrich) in DMSO. Control reactions contained an equal volume of DMSO. In addition, boiled (for 5 min) membranes were assayed to ensure that active transport occurred and the data obtained were not due to entrapment of the phenolic substrate on the surface of the vesicles. Reactions were stopped by the addition of 400–800 µl ice-cold reaction buffer and then gently mixed. Vesicles were pelleted by centrifugation at 17 000 *g* for 5 min at 4 °C, and the supernatant was removed. In the case of ^14^C-substrates, the pellet was suspended in 180 µl water, mixed with 3 ml scintillation liquid (Hisafe 3, Perkin Elmer), and the radioactivity was counted for 5 min in a liquid scintillation counter (Wallac 1415, LSC, Wallac Oy, Turku). The accumulation of coniferin (%) in the vesicles was calculated by dividing the radioactivity of the pellet by the total radioactivity in the reaction and multiplying by 100. In the case of non-radioactive substrates, the pellet was extracted twice for 15 min with 300 µl of 67% methanol supplemented with fluorocinnamic acid as an inner standard by vortexing and sonicating at room temperature. The membranes were pelleted by centrifugation at 19 000 *g* for 10 min at room temperature, after which the supernatant was dried. The sample was dissolved in 60 µl of 100% methanol, and injected for HPLC (Agilent 100 Series; column: ZORBAX RX-C18, 5 µm, 4.6 × 250 mm; G1315B DAD detector), or UPLC (Acquity Ultra Performance LC; column: HSS T3, 1.8 µm, 2.1 × 150 mm; PDA eλ detector). The peak areas of the compounds studied and of the inner standard were integrated and used to quantify the compounds. 

### Statistical analysis

Each experiment was conducted at least three times with different MF preparations, with a similar trend in results. Kruskal–Wallis or one-way ANOVA tests followed by post-hoc tests with Bonferroni correction were performed for coniferin transport results with multiple inhibitors or alternative substrates within one experiment to test the significance of inhibition. Owing to occasional variation in the detection level, both the statistical testing and the representation of results were done from either representative experiments or from data combined from several experiments. A one-tailed Student’s *t*-test was used for pairwise comparison of coniferin and *p-*coumaryl alcohol glucoside cross-inhibition. Kruskal–Wallis tests were performed in SPSS (IBM) and *t*-tests in Excel (Microsoft).

### Sample preparation for proteomic analyses

Sodium deoxycholate (SDC) was used to solubilize membrane proteins from washed UP and MF fractions of the spruce materials (developing xylem, phloem, and cultured cells). All materials had three biological replicates except phloem UP, which had two biological replicates. As the UP samples had more PM protein identifications than the MF samples (see Supplementary Table S1A), the UP samples were additionally solubilized with sodium dodecyl sulfate (SDS) to increase the number of identifications. Protein solubilizations were done in 25 mM Tris–HCl, pH 6.8, supplemented with 25 mM DTT and Complete protease inhibitors (Roche) by adding the detergent dropwise to samples to a 4% final concentration and then incubating the samples at 60 °C for 30 min. The buffer for SDS solubilization was supplemented with 5% glycerol and 0.02% bromophenol blue. The remaining membranes were pelleted by centrifugation at 17 000 *g* for 30 min at room temperature, and the supernatant was collected. The SDS-treated samples were allowed to enter the top part of a polyacrylamide gel in electrophoresis (PAGE), the gel was stained with PageBlue (Bio-Rad), and the area containing proteins was cut out. The gel was destained during the clean-up step using the C18 STAGE-tip (made as described in Obudulu *et al.*, 2016). In the case of SDC-solubilized samples, no PAGE step was done as SDC is compatible with trypsin digestion and MS (Masuda *et al.*, 2008). Proteins were digested with trypsin according to a published procedure (Masuda *et al.*, 2008), followed by a post-digestion clean-up on an Oasis HLB µElution plate (Waters, MA, USA). The eluates were diluted 50-fold to decrease the organic content to ~1% before MS analysis.

### LC-MS/MS analysis

A 1 ng aliquot of each sample was loaded on a BEH C18 analytical column (75 μm internal diameter × 250 mm, 1.7 μm particles; Waters, MA, USA), and separated using a concave 180 min gradient of 1–40% solvent B (0.1% formic acid in acetonitrile) in solvent A (0.1% aqueous formic acid) at a flow rate of 368 nl min^–1^. The eluate was passed to a nano-electrospray ionization-equipped SynaptTM G2-Si HDMS mass spectrometer (Waters, MA, USA) operating in a resolution mode. All data were collected using ion-mobility-MS^E^ with dynamic range extension enabled using a scan time of 0.4 s, mass-corrected using Glu-fibrinopeptide B and Leu-enkephalin as reference peptides.

### Data processing

The data were processed with Protein Lynx Global Server v.3.0.3 (Waters, MA, USA), and the resulting spectra were searched against the *Picea abies* 1.0 database [ConGenIE (http://congenie.org/); Sundell *et al.*, 2015]. The database search settings were: enzyme-specific cleavage with one miscleavage allowed; carbamidomethylated cysteines as fixed modification; oxidized methionine, N-terminal acetylation, and deamidated asparagine and glutamine as variable modifications. A minimum of three fragments were required for a peptide detection with a precursor and fragment tolerance of 10 and 25 ppm, respectively, with a false discovery rate <5%.

### MS data analysis

In the case of UP samples, results from SDS- and SDC-solubilized samples were combined. In the case of MF samples, results from a run with SDC-solubilized samples were used. A protein was identified in the sample if at least one unique peptide was detected, and in the membrane preparation if a protein was found in at least two out of three biological replicates. Sometimes the MS peak was also visible for the third replicate but identification by MS/MS could not be done. In addition to qualitative data, the MS^E^ mode used produced quantitative data. Final lists of proteins were compared with each other and with gene expression correlation results by using Venny 2.1 (http://bioinfogp.cnb.csic.es/tools/venny/). About 30% of Norway spruce genes are fragmented, and the confidence level (http://congenie.org/; Nystedt *et al.*, 2013) is marked in the results presented in Supplementary Table S1.

### Correlation analysis of pre-existing gene expression data

Data from pre-existing gene expression studies in Norway spruce were used to search for suitable monolignol transporter candidates. The datasets were: (i) ConGenIE (http://congenie.org/;Nystedt *et al.*, 2013), 22 libraries from different organs and different time points of the whole aerial part of a tree; (ii) Norwood (Jokipii-Lukkari *et al.*, 2017), 51 libraries of thin layers cut from phloem and cambium over the developing xylem into the mature xylem; (iii) Ray-tracheid comparison (Blokhina *et al.*, 2019), nine libraries comprising developing ray parenchymal cells and tracheids separately, and whole sections of developing xylem; (iv) Tissue culture (Laitinen *et al.*, 2017), 18 libraries of lignin-forming cultured cells under lignin-forming and non-lignin-forming conditions. In a recent study, gene expression in xylem throughout the year was examined and a highly correlating set of 12 monolignol biosynthesis genes with a peak of expression during xylem development was identified (Jokipii-Lukkari *et al.*, 2018). These genes were used here as baits (denoted hereafter as bait genes; Table 1). Pearson’s correlation coefficient was used (in Excel) to search for genes whose expression correlated with that of any of the 12 bait genes with a coefficient >0.8. Each of the four datasets was studied separately. A similar correlation analysis was done using ABC and MFS transporters detected in the proteomic analysis, to gain data on possible co-expression of the transporters with other lignification-related genes. Genes whose expression correlated with that of a selected transporter with a correlation coefficient >0.8 in at least two out of four datasets were selected to represent correlation.

**Table 1.** *K*_m_ values determined for coniferin transport in microsomal fractions prepared from developing xylem and lignin-forming cultured cells of Norway spruce, and BY-2 cells of tobacco

	*K*_m_ (µM)^*a*^	
	Mean	SD
Norway spruce xylem	127.4	21.4
Norway spruce cultured cells	463.1	297.0
BY-2 cells	39.0	0.08

^*a*^ The Lineweaver–Burk equation (Lineweaver and Burk, 1934) was used to calculate *K*_m_.

## Results

### Transport of monolignol glucosides

MF were prepared from developing xylem, phloem, and lignin-forming cultured cells of Norway spruce, and from tobacco BY-2 cells, to test the hypothesis that there are PM-localized, active transporters for monolignol transport. MF were used in the transport assays instead of PM-enriched fraction (UP) for the following reasons. (i) The preparation of MF is much faster than PM enrichment by aqueous polymer two-phase partitioning; consequently, the enzyme activity is better maintained during isolation of the membranes. In both the MF and the PM-enriched fractions of Norway spruce, PM and tonoplast vesicles are present (Kärkönen *et al.*, 2014; Väisänen *et al.*, 2018). Additional methods, such as sucrose gradient centrifugation, would be needed for the preparation of pure PM fractions, extending the isolation time further. (ii) Generally, MF contains both inside-out and right-side-out vesicles of PM, whereas PM-enriched fractions prepared by aqueous polymer two-phase partitioning contain mainly right-side-out vesicles (Larsson *et al.*, 1994), and a method for turning the vesicles inside out would be necessary, and would extend the preparation time for the vesicles. Inside-out vesicles are needed because the method investigates the uptake of substrates into the vesicles. (iii) Isolating PM from conifers such as Norway spruce is much more demanding than from angiosperm species (Kärkönen *et al.*, 2014; Väisänen *et al.*, 2018). The yields of PM-enriched fractions are low and would allow only a limited number of transport assays. (iv) Although MF contains all cellular membranes, the results with transporter assays were similar to those with enriched PM vesicles (data not shown).

**Fig. 2.**
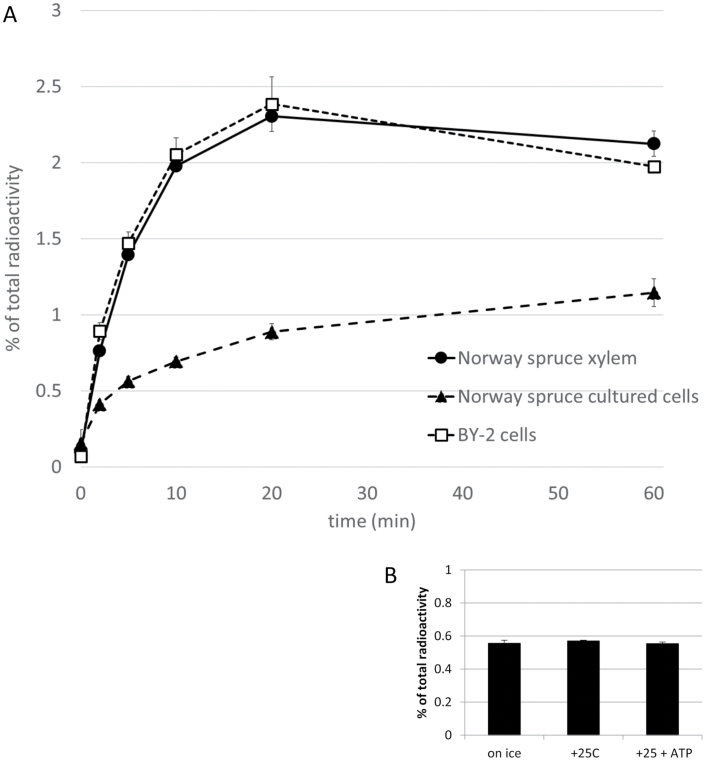
(A) Time course of ATP-dependent ^14^C-coniferin uptake to microsomal vesicles prepared from Norway spruce developing xylem, lignin-forming cultured cells, and tobacco BY-2 cells. The background with no ATP addition was subtracted. (B) ^14^C-coniferin uptake to microsomal vesicles prepared from Norway spruce developing phloem at 0 °C (on ice), and at 25 °C without and with ATP supplementation; time interval 10 min. Data presented are means ±SD, *n*=3.

An ATP-dependent accumulation of ^14^C-coniferin with saturation kinetics was observed in MF prepared from spruce xylem and lignin-forming cultured cells, and in MF prepared from tobacco BY-2 cells (Fig. 2A). However, no transport was observed in vesicles prepared from spruce phloem (Fig. 2B). 

**Fig. 3.**
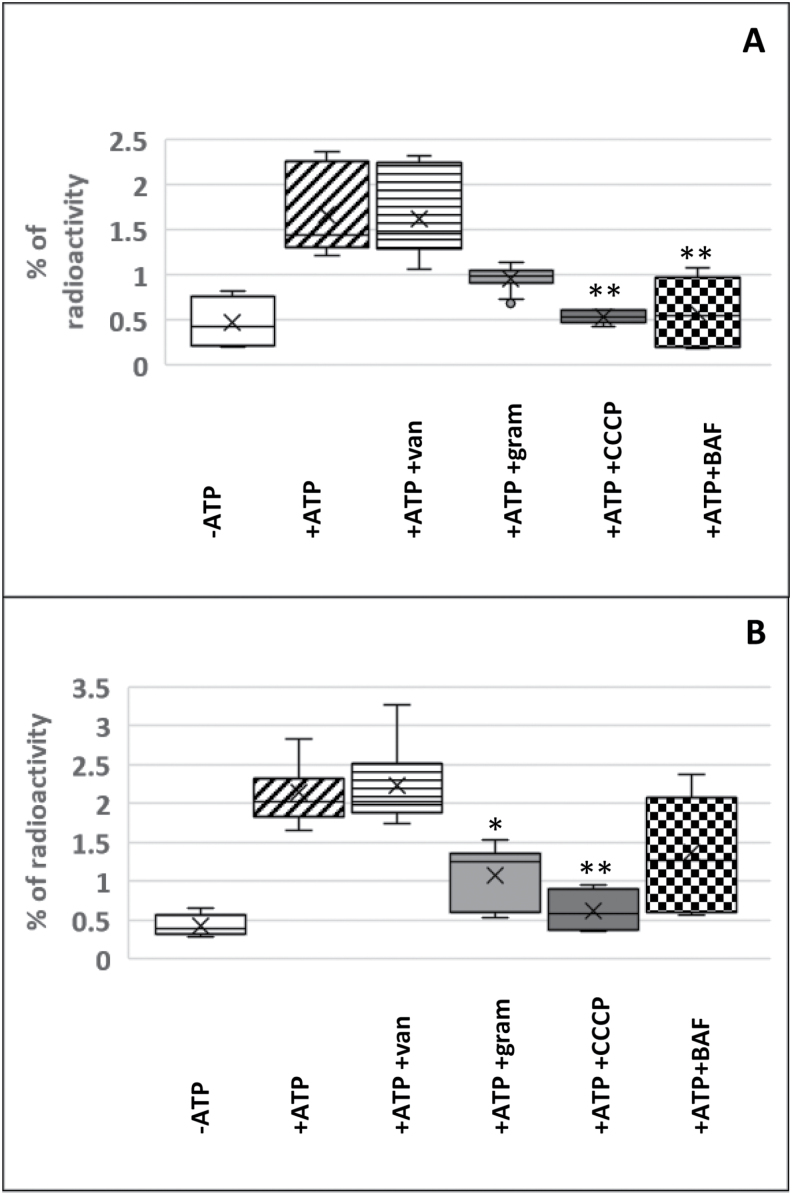
Inhibitor profile of ^14^C-coniferin transport in microsomal vesicles (MF) prepared from (A) Norway spruce developing xylem and (B) tobacco BY-2 cells. +/– 5 mM ATP, +/– 1 mM vanadate (van), +/– 50 µM gramicidin (gram), +/– 20 µM CCCP, and +/– 1 µM bafilomycin A1 (BAF) were used in 10 min reactions. In MF of xylem, transport was significantly inhibited by CCCP (*P*=0.002) and bafilomycin A1 (*P*=0.002) [Kruskal-Wallis test followed by Bonferroni correction (H=26.7, *P*<0.000; combination of three experiments, *n*=6–9)]. In MF of BY-2 cells, transport was significantly inhibited by gramicidin (*P*=0.014) and CCCP (*P*=0.002) [Kruskal-Wallis test followed by Bonferroni correction (H=24.9, *P*<0.000; combination of three experiments, *n*=6–12)].

Next, we tested whether the accumulation of ^14^C-coniferin in spruce xylem and tobacco BY-2 cell MF preparations was inhibited by chemicals that are known to inhibit membrane transport (Fig. 3). With ion uncouplers, it is possible to inhibit a secondary active transport where a transporter is using an ion gradient (in plants, a H^+^ gradient) to energize the transport of its substrate across the membrane. Gramicidin creates an ion channel for monovalent cations (Hladky and Haydon, 1972), while the weak acid CCCP transports H^+^ across the membrane (McLaughlin and Dilger, 1980). To inhibit transport more directly, we also used vanadate and bafilomycin A1. Vanadate inhibits ABC transporters (Urbatsch *et al.*, 1995) as well as the PM H^+^-ATPase (Gallagher and Leonard, 1982), whereas bafilomycin A1 inhibits V-type H^+^-ATPase present in the tonoplast (Dröse and Altendorf, 1997). Both gramicidin and CCCP significantly reduced ^14^C-coniferin transport in MF prepared from BY-2 cells (Fig. 3). In developing xylem, transport was significantly inhibited by CCCP and bafilomycin A1 (Fig. 3). With bafilomycin A1 in BY-2 cells and gramicidin in xylem, a trend towards inhibition was evident, which further indicates a role for a vacuolar coniferin/H^+^ antiporter.

Of all material tested, the transporter in BY-2 vesicles had the highest affinity for coniferin, with a *K*_m_ value of 39.0 µM (Table 1). The *K*_m_ value for coniferin in xylem was three times higher (127.4±21.4 µM), whereas that in spruce cultured cells was the highest (463.1 ±297.0 µM).

**Fig. 4.**
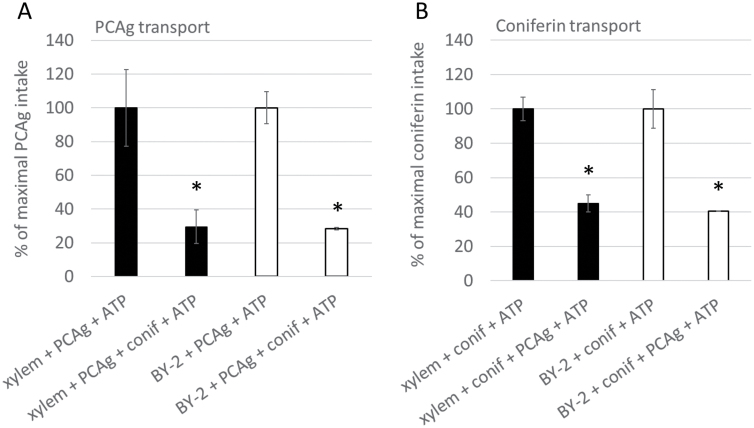
Inhibition of ATP-dependent uptake of (A) *p*-coumaryl alcohol glucoside (PCAg) and (B) ^14^C-coniferin (conif) to microsomal vesicles prepared from Norway spruce developing xylem (black bars) and tobacco BY-2 cells (white bars). Inhibition was achieved with equal concentrations (100 µM) of substrates in the reaction mixture. Data presented are means ±SD, *n* = 3. Asterisks indicate statistically significant transport inhibition (*P*<0.05; one-tailed Student’s *t*-test).

Similarly, ATP-dependent *p*-coumaryl alcohol glucoside transport was observed in MF prepared from developing spruce xylem and tobacco BY-2 cells (Fig. 4A). The inhibition of coniferin transport with an equal concentration of *p*-coumaryl alcohol glucoside, and similar inhibition of *p*-coumaryl alcohol glucoside transport with an equal concentration of coniferin, suggests that the same transporter is transporting these compounds (Fig. 4, Supplementary Fig. S1). Coniferin and *p*-coumaryl alcohol glucoside were the only detected substrates for the transport (Table 2) in developing xylem and BY-2 cells, although pinoresinol was able to inhibit coniferin transport in developing xylem (Supplementary Fig. S1). Other phenolics did not have a statistically significant inhibitory effect on coniferin transport. Coniferin transport in vesicles of developing xylem (mean ±SD 1.45±0.05) was inhibited by the addition of *p*-coumaryl alcohol glucoside (1.08±0.03, *P*=0.000), pinoresinol (1.18±0.06, *P*=0.000), and coniferin (0.93±0.02, *P*=0.000), as revealed by one-way ANOVA [*F*(7, 16)=38.47, *P*=0.000] followed by Bonferroni post-hoc tests (Supplementary Fig. S1). Coniferin transport in BY-2 vesicles was significantly inhibited by *p*-coumaryl alcohol glucoside and coniferin (Supplementary Fig. S1).

**Table 2.** Transport of compounds investigated in microsomal vesicles (MF) prepared from developing xylem of Norway spruce

	–ATP		+ATP		Technical replicates
	Mean	SD	Mean	SD	
Coniferyl alcohol	100	18.1	99.0	20.0	2
Coniferin	100	0.9	**214.1**	10.2	2
*p*-Coumaryl alcohol	100	4.1	94.6	16.2	3
*p*-Coumaryl alcohol glucoside	100	31.6	**342.2**	69.5	3
Pinoresinol	100	1.0	93.0	2.1	3
Pinoresinol glucoside	100	7.5	74.1	5.8	3
Lariciresinol	100	9.8	94.6	6.5	3
Isolariciresinol	100	2.8	88.1	1.8	3
β-*O*-4 erol	100	2.1	107.5	10.0	3

Experiments for each compound were conducted at least twice with a similar trend in results. A representative experiment with the indicated number of technical replicates is shown. As the aim was to resolve whether there was ATP-dependent accumulation of these substrates in MF, and not to compare the accumulation of different substrates with each other, the results are shown relative (%) to the background samples with no ATP supplementation.

No ATP-dependent ^14^C-coniferyl alcohol transport was observed in any of the tested materials (Table 2 and data not shown). These results negate the primary hypothesis that the transported form of monolignol is coniferyl alcohol in developing xylem, similar to that in Arabidopsis leaves (Miao and Liu, 2010). Variations of the reaction mixture [additional salt supplementation (KCl, MgSO_4_, CaCl_2_, or NaCl), xylem harvesting time (early June or late June), different concentrations of coniferyl alcohol, or the use of enriched plasma membranes (UP fraction) instead of MF] did not change this conclusion (data not shown).

No active transport of *p*-coumaryl alcohol or the tested dimers pinoresinol, pinoresinol monoglucoside, lariciresinol, isolariciresinol, and β-*O*-4 erol was detected in xylem MF (Table 2).

### Spruce membrane proteome

The membrane proteomes of developing xylem, phloem, and lignin-forming cultured cells of Norway spruce (three replicates of each) were analyzed to identify candidates for monolignol transport. SDC solubilization of MF and UP proteins led to the identification of 522 and 496 proteins in xylem UP and MF, respectively (Supplementary Table S1A). Specifically, 195 proteins were present only in the UP fraction and 169 were present only in the MF. The proteins specific for the UP fractions (Supplementary Fig. S2A) had, among others, a lot of PM and tonoplast protein identifications, whereas proteins present only in the MF (Supplementary Fig. S2B) were largely of mitochondrial origin. Thus, xylem UP fractions were selected to be analyzed in more detail and were additionally solubilized with SDS to enable more identifications. The protein detections of the SDC- and SDS-solubilized UP fractions and SDC-solubilized MF fractions are shown in Supplementary Table S1B, and their peptides are shown in in Supplementary Tables S2 and S3.

**Fig. 5.**
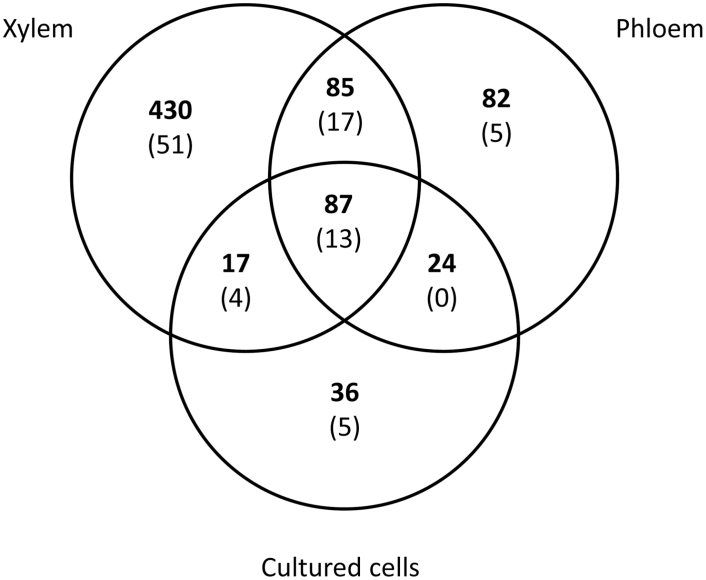
Venn diagram representing protein identifications in the SDC- and SDS-solubilized enriched plasma membrane fractions of Norway spruce developing xylem, phloem, and lignin-forming cultured cells. The number of transporters is given in parentheses.

Altogether, 761 proteins were detected in the UP samples, of which 619 were detected in xylem, 278 in phloem, and 164 in the lignin-forming cultured cells (Fig. 5, Supplementary Table S1B). A REVIGO treemap of proteins in xylem UP showed multiple biological functions, out of which the most obvious was “transport processes” (Supplementary Fig. S3A). The functions “response to different signals” and “involvement of metabolism and biosynthesis” were also abundant. Among phloem UP proteins, “responses to different signals” and “metabolic processes”, especially “carbohydrate metabolism”, were evident, as was “transport” (Supplementary Fig. S3B). The UP fraction of the lignin-forming cultured cells showed functions in similar biological processes, but “transport” was poorly represented (Supplementary Fig. S3C).

Of all proteins identified in the materials analyzed, 95 were transporters (channels, secondary and primary active transporters, and carriers), of which 85 were present in xylem, 35 in phloem, and 22 in the lignin-forming cultured cells (Fig. 5, Supplementary Table S1B). There were 13 transporters common to all the tissues studied. Among these were one ABC transporter, several ATPases/synthases and other proteins for H^+^ translocation, one aquaporin, one ATP/ADP carrier, and one Na^+^/Ca^2+^ exchanger. Four transporters were shared between the two lignin-producing materials (i.e. developing xylem and cultured spruce cells): two H^+^-ATPases, a Ca^2+^-ATPase, and a phosphate transporter. Fifty-one transporters were detected solely in the xylem samples. Of these, 15 were ABC transporters and three were major facilitator superfamily (MFS) transporters. The rest of the transporters were involved in H^+^ translocation, water and ion movement, and other transport processes. Xylem and phloem shared 17 transporters that were not detected in the tissue-cultured cells. These comprised four ABC transporters, two ATP synthases, four H^+^ pumps, three ion channels, three aquaporins, and one homolog of Arabidopsis Walls Are Thin 1 (WAT1, an auxin transporter; Ranocha *et al.*, 2013). Five transporters were found solely in the cultured cells: an ABC transporter, a MFS transporter, two ion channels, and a xanthine/uracil permease family protein. Five transporters were present in phloem only, comprising three aquaporins, one Ca^2+^-ATPase, and one ion channel (Supplementary Table S1B). No multidrug and toxic compound extrusion (MATE) transporters were present in any of the materials. However, a single UP sample obtained from the lignin-forming cultured cells showed two unique peptides with MATE transporter annotation (MA_9267g0020).

### Genes correlating with bait monolignol biosynthesis genes

**Fig. 6.**
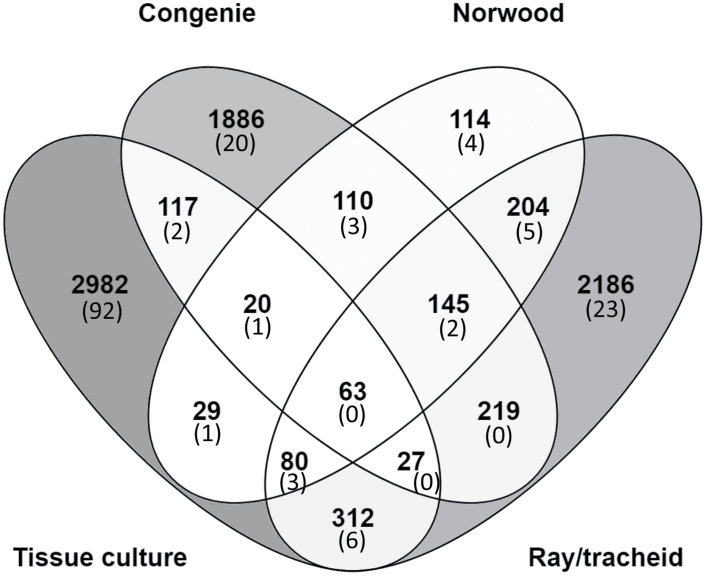
Venn diagram representing genes whose expression correlated with the bait genes in four different datasets: Tissue culture (Laitinen *et al.*, 2017), ConGenIE (Nystedt *et al.*, 2013), Norwood (Jokipii-Lukkari *et al.*, 2017), and Ray-tracheid (Blokhina *et al.*, 2019). The number of ABC, MATE, and MFS transporters is given in parentheses.

The expression patterns of genes belonging to the same developmental trait can correlate, and strong gene co-expression has been detected between a transporter and the biosynthesis genes of its substrate (Darbani *et al.*, 2016). Thus, we conducted a correlation analysis between selected monolignol biosynthesis genes and published gene expression datasets for Norway spruce. All-year-around gene expression data of developing spruce xylem (Jokipii-Lukkari *et al.*, 2018) showed a clear correlation of 12 monolignol biosynthesis genes covering all the enzymatic reactions in the pathways leading to monolignols (Table 3), with maximum expression in the summer months during active xylem development. The correlation of these monolignol biosynthesis genes (hereafter termed “bait genes”) in the published datasets was examined, and they were noticed to correlate positively with each other in all datasets (Supplementary Table S1C). Next, the expression of other genes in relation to the bait genes was analyzed. In the datasets analyzed, ~5% of the genes correlated with the baits with a correlation coefficient of >0.8. Altogether, 8494 genes had a correlation coefficient >0.8 with the bait genes in at least one of the datasets (Supplementary Table S1B), of which 765 were present in the Norwood data (Jokipii-Lukkari *et al.*, 2017), 3630 in the tissue culture data (Laitinen *et al.*, 2017), 3236 in the ray-tracheid data (Blokhina *et al.*, 2019), and 2587 in the ConGenIE data (Nystedt *et al.*, 2013). The gene lists showed some overlap (Fig. 6).

**Table 3.** Genes correlating positively (>0.8) with the bait genes in all four datasets studied, and their presence in the membrane proteomic data of developing xylem, phloem, and lignin-forming tissue-cultured cells of Norway spruce

Gene accession	Annotation	Closest Arabidopsis homolog	Presence in the proteomics data					
			Xylem UP	Phloem UP	Cultured cells UP	Xylem MF	Phloem MF	Cultured cells MF
**Monolignol biosynthesis and assisting reactions**								
MA_10432099g0010	Caffeic acid *O*-methyltransferase *	AT5G54160.1	X			X		
MA_106573g0010	Hydroxycinnamoyl-CoA shikimate/quinate hydroxycinnamoyl transferase*	AT5G48930.1						
MA_109548g0010	*p*-Coumarate 3-hydroxylase *	AT2G40890.1	X			X		
MA_123220g0010	Phenylalanine ammonia lyase *	AT3G53260.1	X		X			
MA_130482g0010	Cinnamate 4-hydroxylase *	AT2G30490.1	X			X		
MA_202753g0010	Cinnamate 4-hydroxylase	AT2G30490.1				X		
MA_362678g0010	Caffeoyl-CoA *O*-methyltransferase *	AT4G34050.1						
MA_52972g0010	Cinnamyl alcohol dehydrogenase *	AT3G19450.1						
MA_56692g0010	4-Coumarate: CoA ligase *	AT3G21240.1						
MA_667858g0010	Caffeoyl-CoA *O*-methyltransferase	AT4G34050.1	X					
MA_6931g0010	Caffeoyl-CoA *O*-methyltransferase *	AT4G34050.1	X					
MA_9446650g0010	Cinnamoyl-CoA reductase *	AT1G15950.1						
*(MA_118702g0010)*	(Cinnamate 4-hydroxylase *)	*AT2G30490.1*	*X*		*X*	*X*		
*(MA_87599g0010)*	(Caffeoyl shikimate esterase *)	*AT1G52760.1*						
**Shikimate pathway**								
MA_10428955g0020	Chorismate synthase 2	AT1G48850.1						
MA_10436001g0020	Phospho-2-dehydro-3-deoxyheptonate aldolase 1	AT1G22410.1	X			X		
MA_107600g0010	3-Dehydroquinate synthase	AT5G66120.2						
MA_43667g0010	Arogenate dehydratase/prephenate dehydratase 6	AT1G08250.1						
MA_4908g0010	3-Dehydroquinate synthase	AT5G66120.2						
MA_76465g0010	3-Phosphoshikimate 1-carboxyvinyltransferase	AT2G45300.1						
MA_8419g0010	Aspartate aminotransferase	AT2G22250.2						
MA_8918g0010	Chorismate synthase	AT1G48850.1						
**Redox enzymes**								
MA_125690g0020	Flavoprotein WrbA	AT4G27270.1	X					
MA_21175g0010	Cytochrome b5 isoform B	AT2G32720.1	X		X	X	X	
MA_76916g0010	Thioredoxin superfamily protein	AT5G38900.1						
MA_8687g0010	NADH-cytochrome b5 reductase 1	AT5G17770.1	X			X	X	
**Transport**								
MA_1461g0010	Putative cadmium/zinc-transporting ATPase HMA4	AT4G30110.1						
MA_18076g0010	Amino acid permease 3	AT1G77380.1						
MA_198319g0010	H^+^-ATPase interacting protein	AT4G27500.1						
MA_84518g0010	Copper-transporting ATPase RAN1	AT5G44790.1						
**Kinases**								
MA_118589g0010	Probable receptor-like protein kinase	AT3G59110.1	X					
MA_121123g0010	Proline-rich receptor-like protein kinase PERK1	AT1G77280.1						
MA_255g0010	Adenosine kinase 2	AT5G03300.1	X	X	X			
**Transcription factors**								
MA_10434782g0020	LOB domain-containing protein 6/AS2	AT1G65620.1 (AS2)						
MA_33964g0010	Protein ODORANT1/MYB	AT5G16600.1 (MYB43)						
**Cell wall polysaccharide synthesis or modification, and sugar metabolism**								
MA_10425819g0010	Beta-glucosidase 42	AT5G36890.1						
MA_10427170g0020	Sucrose synthase 4	AT4G02280.1	X			X		
MA_10429529g0010	Probable glucuronoxylan glucuronosyltransferase IRX7	AT2G28110.1						
MA_10433350g0010	Glycosyltransferase family protein (DUF23)	AT2G33570.1						
MA_246547g0010	Callose synthase 3	AT5G13000.1						
MA_71720g0010	Mannan endo-1,4-β-mannosidase 2	AT2G20680.1						
MA_10433720g0010	DUF246 domain-containing protein	AT1G62330.1						
**Amino acid biosynthesis and metabolism (other than aromatic amino acids)**								
MA_10090g0010	5-Methyltetrahydropteroyltriglutamate-homocysteine methyltransferase	AT5G17920.1	X	X	X	X		X
MA_10427606g0010	5-Methyltetrahydropteroyltriglutamate-homocysteine methyltransferase	AT5G17920.1	X			X		
MA_10430803g0010	4,5-DOPA dioxygenase extradiol-like protein	AT4G15093.1						
MA_10435905g0030	D-3-Phosphoglycerate dehydrogenase	AT4G34200.1						
MA_11357g0010	Serine hydroxymethyltransferase 1	AT4G13930.1	X	X	X	X		
MA_17826g0020	Phosphoserine aminotransferase	AT4G35630.1						
**Lipid synthesis and modification**								
MA_177103g0010	Sec14p-like phosphatidylinositol transfer family protein	AT3G24840.1						
MA_803706g0010	Acyl-CoA-binding domain-containing protein 4	AT3G05420.2						
**Microtubule-associated**								
MA_10436304g0020	TPX2 (targeting protein for Xklp2) protein family	AT2G35880.1						
MA_7104g0010	GPI-anchored adhesin-like protein, putative (DUF936)	AT1G08760.1						
**Metabolism**								
MA_101067g0010	Fructose-bisphosphate aldolase	AT3G52930.1	X	X	X	X		X
MA_10431598g0010	ATP-citrate synthase alpha chain protein 3	AT1G09430.1						
MA_11783g0010	Probable glycine cleavage system H protein 2	AT2G35120.1						
MA_197296g0010	*S*-adenosylmethionine synthase 3	AT3G17390.1	X	X	X	X		
MA_736502g0010	Pyrophosphate-fructose 6-phosphate 1-phosphotransferase subunit alpha	AT1G76550.1						
MA_81112g0010	Methylenetetrahydrofolate reductase 1	AT2G44160.1	X	X				
MA_9153293g0010	Alcohol dehydrogenase class-3	AT5G43940.2						
**Other**								
MA_10429738g0010	Maternal effect embryo arrest 59	AT4G37300.1						
MA_138523g0010	Chaperone protein DnaJ	AT2G22360.1						
MA_23219g0010	Cell number regulator 8	AT2G37110.1						
MA_70960g0010	AAR2 protein family	AT1G66510.1						
MA_87543g0010	Uncharacterized protein C4orf29 homolog	AT3G12150.1						
MA_9061045g0010	NA							

Twelve monolignol biosynthesis genes (Jokipii-Lukkari *et al.*, 2018) that were used as bait genes in a correlation study are marked with asterisks. Two bait genes (*Cinnamate 4-hydroxylase*, MA_118702g0010; *Caffeoyl shikimate esterase*, MA_87599g0010; indicated in parentheses) had positive correlations with the other baits, but the value did not exceed 0.8 in all datasets. UP, Enriched plasma membrane vesicles; MF, microsomal vesicles; NA, no annotation.

Sixty-three genes correlated with the baits with correlation coefficients >0.8 in all datasets studied, creating a candidate list of genes related to lignin biosynthesis (Table 3). Although all 12 bait genes had positive correlations with each other, the correlation coefficients of two genes did not exceed 0.8; these are included in Table 3. Of the genes shown in Table 3, 20 were present in the proteomic data of the xylem samples. Among the genes, there were two additional monolignol biosynthesis genes (C4H, MA_202753g0010; CCoAOMT, MA_667858g0010). Three transporters (amino acid permease, copper-transporting ATPase, and putative cadmium/zinc-transporting ATPase) correlated with the baits, along with H^+^-ATPase interacting protein (Table 3). None of these were detected in the proteomic data. The shikimate pathway was represented by eight members. Four redox enzymes were also detected. Three kinases, a transcription factor LOB domain-containing protein 6, and a MYB transcription factor also had good co-expression with the baits.

### Identifying candidate transporters from the proteomic and correlation data

We used four criteria in our search for candidates for monolignol transporter(s). (i) ABCB and ABCG transporters were preferred due to their reported monolignol transport and similar known substrates (Yazaki, 2006; Rea, 2007; Verrier *et al.*, 2008; Yazaki *et al.*, 2008; Alejandro *et al.*, 2012), and MFS and MATE transporters were preferred due to the detected coniferin/H^+^-antiporter activity in the Norway spruce xylem in the present study (Fig. 3). (ii) The correlation of the monolignol biosynthesis bait genes with transporters (see above). (iii) The presence of a transporter in the proteomic data, and its correlation in gene expression with lignification-related genes. (iv) In the case of ABC transporters, sequence similarity to the putative *p*-coumaryl alcohol transporter AtABCG29 (Alejandro *et al.*, 2012).

In the gene correlation analysis, 162 ABC, MFS, or MATE transporters showed co-expression with the bait genes in at least one of the datasets used (Fig. 6, Supplementary Table S1B). Six transporters showed co-expression with the baits in three datasets (one ABC transporter and five MFS transporters), and 17 transporters had co-expression with the baits in two datasets. Of these, several ABCB, ABCG, and MFS transporters, and two MATE transporters were considered as candidates (Table 4). Six ABC transporters and one MFS transporter were detected in both the proteomic and the correlation analyses in at least one dataset (Table 4). An ABCG transporter (MA_18770g0010), which correlated with the baits in three datasets and was detected in the xylem UP fraction, makes a good candidate for monolignol transport. Its sequence homology to the putative Arabidopsis *p*-coumaryl alcohol transporter AtABCG29 (Alejandro *et al.*, 2012) was good (59.6%). Additionally, two spruce proteins with sequence homology to AtABCG29 (MA_135152g0010, 67.6%; MA_10260477g0010, 54.6%) were present in the xylem and phloem UP fractions. A MFS transporter (MA_10428871g0010) whose closest Arabidopsis homolog is annotated as a monosaccharide-sensing protein (AT4G35300.1, 56.9%), was found in the xylem UP vesicles and correlated with the baits in the tissue culture data. Two other transporters, MA_10437245g0020 (transmembrane protein 184A) and MA_10429543g0010 (plastidic glucose transporter 4) had co-expression with the baits in two datasets and were present in developing xylem membranes (Table 4).

**Table 4.** Candidate transporters for monolignol transport in Norway spruce

Gene accession	Subgroup	Uniprot annotation	Closest Arabidopsis homolog	Sequence homology to the closest Arabidopsis homolog (%)	Substrate of the closest Arabidopsis homolog	Presence in the proteomic data						Correlation to baits in gene expression data				Picked based on correlation to other lignin biosynthesis genes
						Xylem UP/MF	Phloem UP/MF	Cultured cells UP/ MF	Xylem MF	Phloem MF	Cultured cells MF	Tissue culture (Laitinen *et al.*, 2017)	Congenie (Nystedt *et al.*, 2013)	Norwood (Jokipii- Lukkari *et al.*, 2017)	Ray- tracheid (Blokhina *et al.*, 2019)	
**ABC transporters**																
MA_10434957g 0010 ^*a*^	B	ABC transporter B family member 1	AT2G36910.1 ABCB1	70.6	IAA	X	X		X				X			X
MA_62683g 0010	B	ABC transporter B family member 1	AT2G36910.1 ABCB1	60.2	IAA	X							X			
MA_138894g 0010	B	ABC transporter B family member 4	AT2G47000.1 ABCB4	65.1	Auxin								X	X		
MA_635039g 0010	B	ABC transporter B family member 11	AT1G02520.1 ABCB11	70.0	?								X	X		
MA_9415070g 0020	B	ABC transporter B family member 15	AT3G28345.1 ABCB15	53.8	?	X							X			
MA_40328g 0010	B	ABC transporter B family member 20	AT3G55320.1 ABCB20	77.0	?	X									X	
MA_107576g 0010	B	Putative multidrug resistance protein	AT3G28345.1 ABCB15	55.7	?									X	X	
MA_10260477g 0010 ^*b*^	G (associated)	Pleiotropic drug resistance 1	AT3G16340.1 ABCG29	54.6	*p*-Coumaryl alcohol	X	X									
MA_135152g 0010 ^*b*^	G	ABC transporter G family member 29	AT1G59870.1 ABCG36	67.2	Cadmium	X	X									
MA_134489g 0020 ^*a,b*^	G	Probable pleiotropic drug resistance protein 2	AT3G16340.1 ABCG29	53.8	*p*-Coumaryl alcohol	X			X							X
MA_17319g 0020 ^*a*^	G	Probable pleiotropic drug resistance protein 2	AT1G15520.1 ABCG40	69.0	ABA	X	X	X	X				X			X
MA_18770g 0010 ^*a*^	G	Putative pleiotropic drug resistance protein 7	AT1G15520.1 ABCG40	66.5	ABA	X						X		X	X	X
MA_31011g 0010 ^*a*^	G	Putative pleiotropic drug resistance protein 7	AT1G15520.1 ABCG40	50.5	ABA							X			X	
***MFS transporters***																
MA_10428182g 0010	MFS	Plastidic glucose transporter 4	AT5G16150.1	75.3	Glucose							X	X			
MA_22713g 0010	MFS	Probable anion transporter 3	AT2G38060.1	57.4	Inorganic phosphate, sugar							X	X	X		
MA_10437216g 0010	MFS	Probable peptide/nitrate transporter	AT3G54450.1	48.6	Oligopeptide?							X		X	X	
MA_10436119g 0010	MFS	Inositol transporter 1	AT2G43330.1	49.5	*Myo*-inositol							X			X	
MA_5112g 0010	MFS	Probable peptide/ nitrate transporter	AT1G22540.1	56.1	Oligopeptide							X			X	
MA_13801g 0010	MFS	Sugar transporter ERD6-like 16	AT5G18840.1	52.5	Carbohydrate								X	X	X	
MA_77652g 0010	MFS	Sugar transporter ERD6-like 16	AT5G18840.1	54.9	Carbohydrate									X	X	
MA_130810g 0010 ^*a*^	MFS	Sugar transport protein 13	AT5G26340.1	74.1	Glucose, hexose			X								X
MA_10428871g 0010	MFS	Monosaccharide- sensing protein 2	AT4G35300.1	56.9	Monosaccharide	X						X				
MA_10429543g 0010 ^*a*^	Sugar (and other) transporter)	Plastidic glucose transporter 4	AT5G16150.1	83.7	Glucose (putative)	X			X			X	X			X
***MATE and other transporters***																
MA_10437152g 0010	MATE	MATE efflux family protein 1	AT1G51340.2	57.2	?							X			X	
MA_94941g 0010	MATE	Protein TRANSPARENT TESTA 12	AT5G44050.1	45.2	?								X	X		
MA_10437245g 0020 ^*a*^	Organic solute transporter Ostalpha	Transmembrane protein 184A	AT3G05940.1	54.7	?	X							X		X	

The presented transporters were detected in the proteomic and/or correlation analysis. Correlation in gene expression to baits in two out of four datasets, or the presence in the proteomic data of lignifying materials combined with correlation in gene expression to the baits or other lignin biosynthesis genes earned the transporter a place in the candidate list. UP, Enriched plasma membrane vesicles; MF, microsomal vesicles. ^*a*^ The best candidates. ^*b*^ Arabidopsis sequence homolog transports *p*-coumaryl alcohol.

Many other transporters were detected in the membrane proteomes of lignifying cell types but did not show co-expression with the baits (Supplementary Table S1B). A correlation analysis was done with these transporters as “baits” to search for co-expression with other lignification-related genes. This analysis led to the identification of an ABCG transporter (MA_134489g0020) and a MFS transporter (MA_130810g0010) (Table 4). The ABCG transporter was detected in developing xylem and correlated with caffeic acid 3-*O*-methyltransferase, a peroxidase, a dirigent protein, and a glucan endo-1,3-β-glucosidase (Supplementary Table S3). The MFS transporter was detected in lignin-forming cultured cells, and correlated in gene expression with three monolignol pathway genes, six glycosyltransferases, five peroxidases, 12 chitinases, and one dirigent protein (Supplementary Table S3).

## Discussion

### Transport of lignin precursors in various materials

Monolignol glucosides are candidates for transported forms of monolignols as they are found in the cambial sap and developing xylem in many gymnosperm tree species (e.g. Freudenberg and Harkin 1963; Schmid and Grisebach, 1982; Savidge, 1989; Savidge and Förster, 1998; Tsuji *et al.*, 2004; Morikawa *et al.*, 2010; Tsuyama and Takabe, 2014; Aoki *et al.*, 2016; Terashima *et al.*, 2016). Monolignol aglycones, by contrast, are not usually detected in metabolite analyses (e.g. Laitinen *et al.*, 2017) or are detected only at low levels (Schmid and Grisebach, 1982; Savidge and Förster, 2001).

Here, we observed coniferin transport in MF prepared from developing xylem and lignin-forming cultured cells of Norway spruce, as well as MF prepared from non-lignin-forming tobacco BY-2 cells (Fig. 2). By contrast, vesicles prepared from developing spruce phloem that does not lignify did not show this transporter activity. Millimolar concentrations (up to 3.7 mM) of coniferin have been detected in the cambial sap of Norway spruce (Schmid and Grisebach, 1982), hence, the *K*_m_ value for coniferin (0.13 mM) in developing spruce xylem determined in our study is in the same range. BY-2 cells do not contain endogenous coniferin, suggesting that the transport activity detected here could be to compartmentalize and detoxify xenobiotics in the vacuole (Le Roy *et al.*, 2016). Biochemical results suggest that the two transporters present in developing spruce xylem and tobacco BY-2 cells (putative orthologs) are tonoplast-located H^+^/coniferin antiporters (e.g. MATE or MFS transporters). This conclusion was deduced from their inhibition by bafilomycin A1 (a tonoplast H^+^-pump inhibitor), gramicidin, and CCCP (both ion gradient uncouplers). Based on *K*_m_ values (Table 1), the coniferin transport detected in spruce xylem and lignin-forming cultured cells was likely to be mediated by different transporters. Thus, Norway spruce seems to have two transporters capable of transporting coniferin, of which the tonoplastic xylem transporter appears to be biochemically similar to the transporters detected in several tree species (Tsuyama *et al.*, 2013) and could even be similar to the coniferin transporter detected by Miao and Liu (2010) in Arabidopsis aerial tissues. Recently, Japanese cypress and hybrid poplar were shown to contain *p*-coumaryl alcohol glucoside transport activity (Tsuyama *et al.*, 2019). Interestingly, coniferin and *p*-coumaryl alcohol inhibited the *p-*coumaryl alcohol glucoside-transporting activity, but only when added in excess (5-fold concentration). The transporters in spruce xylem and tobacco BY-2 cells were also able to transport *p*-coumaryl alcohol glucoside, but the transport was inhibited by an equal concentration of coniferin (Fig. 4, Supplementary Fig. S1). The results obtained in the present study do not support the hypothesis of PM transport of monolignol alcohols, as detected in Arabidopsis (Miao and Liu, 2010; Alejandro *et al.*, 2012), or the hypothesis of PM transport of monolignol dimers or dimer glucosides (Table 2).

Since tobacco BY-2 cells do not lignify or contain coniferin (Väisänen *et al.*, 2015) but have a similar tonoplastic transporter for coniferin as developing xylem cells (Figs 2, 4), it is unlikely that this transporter is specifically involved in lignification. The transporter in BY-2 cells is probably not specific for coniferin and could have other substrate(s) *in vivo*. The same could be true for the transporter detected in developing xylem. If these two proteins are orthologs and serve the same biological function *in planta*, it is possible that they function in stress responses, for example, against pathogens or herbivores, by transporting coniferin to vacuoles for storage, from where it can be released after tissue rupture and hydrolyzed to coniferyl alcohol. Since coniferyl alcohol is toxic (Väisänen *et al.*, 2015), it could serve as a protectant. This could be a ubiquitous feature in plant cells. This idea is supported by the transport results reported by Miao and Liu (2010), who showed that vacuolar vesicles prepared from Arabidopsis aerial parts consisting mostly of non-lignifying mesophyll cells have a biochemically similar coniferin transporter. However, in xylem of gymnosperms, where high amounts of coniferin are present in vacuoles (Morikawa *et al.*, 2010), coniferin (as well as *p*-coumaryl alcohol glucoside) is likely to be the preferred substrate. A model in which coniferin is released from its vacuolar storage, the glucose moiety is cleaved, and the released coniferyl alcohol is used in lignin polymerization is supported by the presence of coniferin β-glucosidases, which have been shown to exist in the xylem of lodgepole pine and poplar (Dharmawardhana *et al.*, 1995; Samuels *et al.*, 2002; Le Roy *et al.*, 2016). Thus, a coniferin transporter could be part of the lignin biosynthesis machinery in xylem. Alternatively, as suggested by Tsuyama et al. (2013, 2019), coniferin and *p*-coumaryl alcohol glucoside transporter(s) may localize in the endomembranes and participate in vesicular transport of monolignols into the cell wall.

### Membrane proteome for Norway spruce developing xylem, phloem, and lignin-forming cells

In order to find candidate transporters for monolignols, we carried out a proteomic analysis of membrane proteins isolated from developing xylem and lignin-forming cultured cells of Norway spruce. As a comparison, developing phloem, where only a small portion of cells lignify (those developing to stone cells), was used. The 619 proteins in the developing xylem UP fraction included enzymes with roles in multiple biological functions. Since one-third of the identifications were of membrane transporters and proteins involved in vesicular transport (Supplementary Table S1B, Supplementary Fig. S3A), the importance of relocation of molecules is obvious. The situation is very similar to that in developing xylem of poplar (*Populus tremula × P. tremuloides*; Nilsson *et al.*, 2010), where secondary cell wall is deposited and vesicular trafficking is needed not only for lignification but also for hemicellulose and cell wall protein deposition.

The 483 proteins present specifically in the UP samples of developing xylem and lignin-forming cultured cells but not in non-lignifying phloem (Fig. 5) include proteins putatively involved in secondary cell wall formation and lignification. The small set of 17 proteins shared between the UP samples of the two lignifying materials support this, since it included two monolignol biosynthesis genes, NADPH-cytochrome P450 reductase, flavoprotein WrbA (a quinone reductase), sucrose synthase, and cytochrome b5 isoform B (Supplementary Table S1B). The xylem UP fraction also included proteins involved in signaling, metabolism, and biosynthesis (Supplementary Fig. S3A).

### Candidates for monolignol transport in Norway spruce

To identify candidate monolignol transporters in Norway spruce, a membrane proteomic analysis of developing xylem, phloem, and lignin-forming cultured cells was complemented with a correlation analysis of pre-existing gene expression data. Genes encoding transporters that were co-expressed with the monolignol biosynthesis genes were tabulated (Table 4, Supplementary Table S1B). The biochemical results support the role of an H^+^ antiporter in monolignol transport, as coniferin and *p*-coumaryl alcohol glucoside were transported into Norway spruce xylem vesicles with this type of transporter (Figs 3, 4), in a similar fashion to that observed in other tree species (Tsuyama et al., 2013, 2019). Thus, MFS transporters that use chemiosmotic gradients for uniport, symport, or antiport functions (Remy and Duque, 2014) are our candidates for monolignol transport. These transporters have previously been associated with the transport of sugars, nitrate, and oligopeptides (Remy and Duque, 2014). In the present study, the expression of multiple spruce MFS transporters was found to correlate with that of the monolignol biosynthesis genes, and two of them were present in the proteomic results (Table 4). Thus, some of these transporters could function in the monolignol glucoside transport that was detected in developing xylem and lignin-forming cultured cells (Figs 2–4). MA_130810g0010 was the only MFS detected in the cultured cells (Table 4, Supplementary Table S1B). Furthermore, this gene showed co-expression with three monolignol biosynthesis genes and six glycosyltransferases (Supplementary Table S3), making it a suitable candidate for the coniferin transporter. The MFS MA_10428871g0010, which was detected in xylem, is another good candidate.

Our results suggest that MATE transporters are not involved in monolignol transport, as no MATE transporters were detected in the membrane proteomic analysis, and the expression of only two MATE transporter genes correlated with that of the monolignol biosynthesis baits. MATE transporters are known to transport a variety of secondary metabolites, such as flavonoids and alkaloids, xenobiotics, citrate, and phytohormones (Takanashi *et al.*, 2014).

ABC transporters transport multiple phenolic substrates (reviewed by Lefèvre and Boutry, 2018), and also hormones (ABCBs in auxin transport and ABCGs in abscisic acid transport). Table 4 includes several ABC transporters that were either found in the membrane proteome of developing xylem/lignin-forming cultured cells or showed co-expression with the monolignol biosynthesis genes. Similar to that reported in Arabidopsis (Kaneda *et al.*, 2011), multiple ABCB transporters in spruce correlated with the monolignol biosynthesis enzymes in terms of gene expression. Out of these transporters, two spruce ABCB transporters were sequence homologs with Arabidopsis ABCB11 and ABCB15, which have been shown to be involved in auxin transport (Kaneda *et al.*, 2011). Six ABCG transporters that have sequence homology to some known or hypothesized monolignol transporters were detected (Table 4). ABCG transporters have been linked to monolignol transport, since substrates of this subgroup include several phenolic compounds (e.g. *p*-coumaryl alcohol; Alejandro *et al.*, 2012; Lefèvre and Boutry, 2018). In addition, multiple Arabidopsis ABCGs correlate in terms of their gene expression with several lignin biosynthesis genes (Takeuchi *et al.*, 2018b), to a transcription factor, MYB58, which is involved in lignin biosynthesis (Zhou *et al.*, 2009), and to a peroxidase, AtPrx25, with a suggested role in lignification during tracheary element differentiation in cell suspension cultures (Takeuchi *et al.*, 2018a). AtABCG36 is a candidate from such a screen (Takeuchi *et al.*, 2018a). AtABCG40 (the closest sequence homolog to three spruce candidates in Table 4), however, is likely to be an abscisic acid transporter (Kang *et al.*, 2010).

### New players for lignin biosynthesis from gene co-expression and proteomic analyses?

The membrane proteome has great potential to reveal new players for lignification, as a number of monolignol biosynthesis enzymes are known to be anchored to membranes (as discussed below). In addition, a new computational study by Vermaas *et al.* (2019) suggests that multiple phenolic compounds involved in lignification can diffuse into or even be enriched in membrane bilayers, thus creating an environment with a high substrate concentration for the enzymes. In our study, the genes correlating in terms of expression with the 12 monolignol biosynthesis bait genes identified by Jokipii-Lukkari *et al.* (2018) are largely involved in monolignol production, and one-third of them putatively function as enzymes bound to a membrane (Table 3). The present results suggest that the co-expressed set of monolignol biosynthesis genes identified by Jokipii-Lukkari *et al.* (2018) is two genes larger and includes C4H (MA_202753g0010) and CCoAOMT (MA_667858g0010). Eight out of these 14 genes (PAL, C3H, three C4Hs, COMT, and two CCoAOMTs) were detected in the membrane proteomic data (Table 3). It is interesting to hypothesize that a proportion of the proteins encoded by the co-expressed genes could function in association with the endoplasmic reticulum (ER), or with other ER proteins, as discussed by Wang *et al.* (2019). C4H/C3H function as homo- and heteromers associated with the ER (Chen *et al.*, 2011; Bassard *et al.*, 2012), and some PAL enzymes associate with C4H (Achnine *et al.*, 2004). COMT and CCoAOMT, on the other hand, have not been shown to localize in the ER, or to form any complexes. Whether the spruce COMT and CCoAOMT now detected interact with membranes or with membrane proteins remains to be resolved. 4CL (Chen *et al.*, 2014) and CCR and CAD (Yan *et al.*, 2019; not detected in the present proteomic results), by contrast, form heterodimers that are putatively cytoplasmic.

Cell wall modification seems to be tightly coordinated with lignification, and the results suggest that the regulation of these pathways is mediated by a transcription factor LOB domain-containing protein/AS2 (MA_10434782g0020), found to co-express with the baits reported in Jokipii-Lukkari *et al.* (2018) and a novel MYB (MA_33964g0010) (Table 3).

## Conclusions

Biochemical transport assays conducted with vesicles isolated from developing xylem, phloem, and lignin-forming cultured cells of Norway spruce point to MFS- or MATE-transporter-mediated monolignol glucoside transport, and do not support such a role for ABC transporters. Xylem vesicles transported both coniferin and *p*-coumaryl alcohol glucoside, but inhibitor assays suggested that this transport is across the tonoplast. The transport of monolignol glucosides was also detected in vesicles prepared from tobacco BY-2 cells, which do not lignify. The presence of monolignol glucoside transport activity in BY-2 cells suggest that the transport activity is not involved in lignification but may be involved in, for example, general defense. Based on *K*_m_ values, lignin-forming, tissue-cultured cells of spruce had a different transporter for coniferin than the transporter in xylem. Comparison of four published gene expression datasets (ConGenIE, Norwood, Norway spruce tissue culture, and Norway spruce ray-tracheid) revealed a number of potential transporters for monolignols/monolignol glucosides. The proteomic and/or co-expression analyses resulted in a list of candidate genes for monolignol/monolignol glucoside transport, consisting of 13 ABC transporters, nine MFS transporters, and four MATE or other transporters. These are candidate transporters for further studies.

## Supplementary data

The following supplementary data are available at *JXB* online.

Protocol S1. Synthesis of ^14^C-coniferin.

Fig. S1. Inhibition of ^14^C-coniferin transport in vesicles prepared from Norway spruce developing xylem and tobacco BY-2 cells with different phenolic compounds.

Fig. 2. REVIGO treemaps showing cellular component of sodium deoxycholate-solubilized upper phase and microsomal fractions of Norway spruce developing xylem.

Fig. S3. REVIGO treemaps showing biological function of sodium deoxycholate- and sodium dodecyl sulfate-solubilized upper phase fractions of Norway spruce developing xylem, developing phloem, and lignin-forming cultured cells.

Table S1. Protein identifications of all membrane fractions and genes correlating with monolignol biosynthesis bait genes.

Table S2. Proteins and peptides detected in the proteomic analysis of Norway spruce UP fractions.

Table S3. Proteins and peptides detected in the proteomic analysis of Norway spruce MF fractions.

eraa368_suppl_Supplementary_Table_1

eraa368_suppl_Supplementary_Table_2

eraa368_suppl_Supplementary_Table_3

eraa368_suppl_Supplementary_Figure

## Data Availability

The mass spectrometry proteomic data have been deposited to the ProteomeXchange Consortium via the PRIDE (Perez-Riverol *et al.*, 2019) partner repository with the dataset identifier PXD017533.
