# Supplementary material for: Hunting monolignol transporters: membrane proteomics and biochemical transport assays with membrane vesicles of Norway spruce
Source: J Exp Bot. 2020 Aug 10;71(20):6379–95. doi: 10.1093/jxb/eraa368 (PMC7586744; doi:10.1093/jxb/eraa368)
Supplement: eraa368_suppl_Supplementary_Figure [file eraa368_suppl_supplementary_figure.pdf]

Hunting monolignol transporter(s): membrane proteomics and biochemical transport assays with membrane vesicles of Norway spruce

Enni Väisänen, Junko Takahashi, Ogonna Obudulu, Joakim Bygdell, Pirkko Karhunen, Olga Blokhina, Teresa Laitinen, Teemu H. Teeri, Gunnar Wingsle, Kurt V. Fagerstedt and Anna Kärkönen

Supplementary Figures S1-S3,

Supplementary Protocol S1

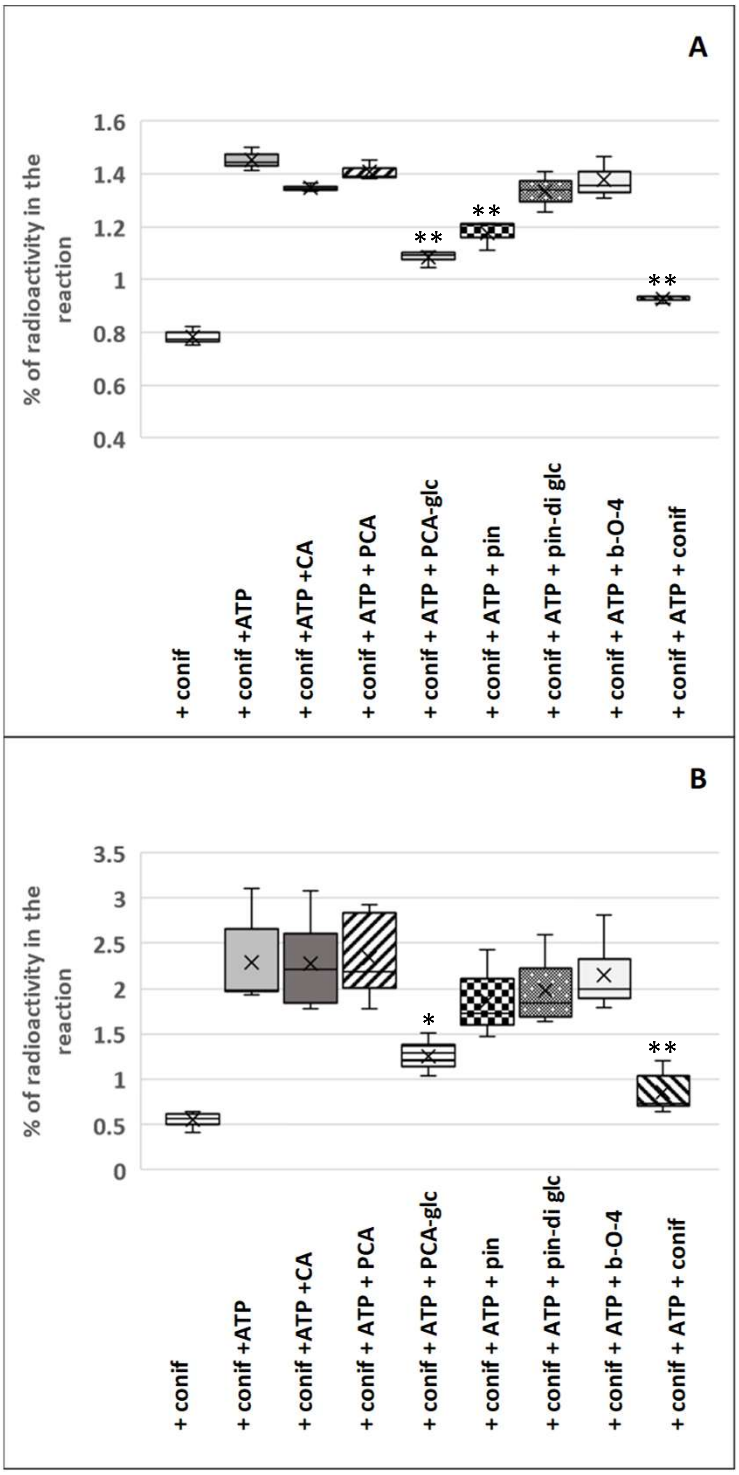

Fig. S1. Inhibition of ^{14}C-coniferin transport in microsomal vesicles (MF) prepared from A) Norway spruce developing xylem, and B) tobacco BY-2 cells with different phenolic compounds. Conif = coniferin, CA = coniferyl alcohol, PCA = *p*-coumaryl alcohol, PCAglc = *p*-coumaryl alcohol 4-O-glucoside, pin = pinoresinol, pin-diglc = pinoresinol diglucoside, b-O-4 = β-O-4 erol. A representative experiment is shown for developing xylem, n = 3. In MF of developing xylem (M 1.45, SD 0.05) transport was significantly inhibited by *p*-coumaryl alcohol glucoside (M 1.08, SD 0.03, *p* 0.000), pinoresinol (M 1.18, SD 0.06, *p* 0.000) and coniferin (M 0.93, SD 0.02, *p* 0.000) as revealed by one-way ANOVA [$F(7, 16) = 38.47$, $p = 0.000$] followed by Bonferroni post-hoc tests. For BY-2 cells, data combined from three individual experiments are shown, n = 7-8. Coniferin transport in BY-2 vesicles was significantly inhibited by *p*-coumaryl alcohol glucoside ($p = 0.013$) and coniferin ($p = 0.001$) as indicated by a Kruskal-Wallis test followed by Bonferoni correction ($H = 37.222$, $p < 0.000$).

A

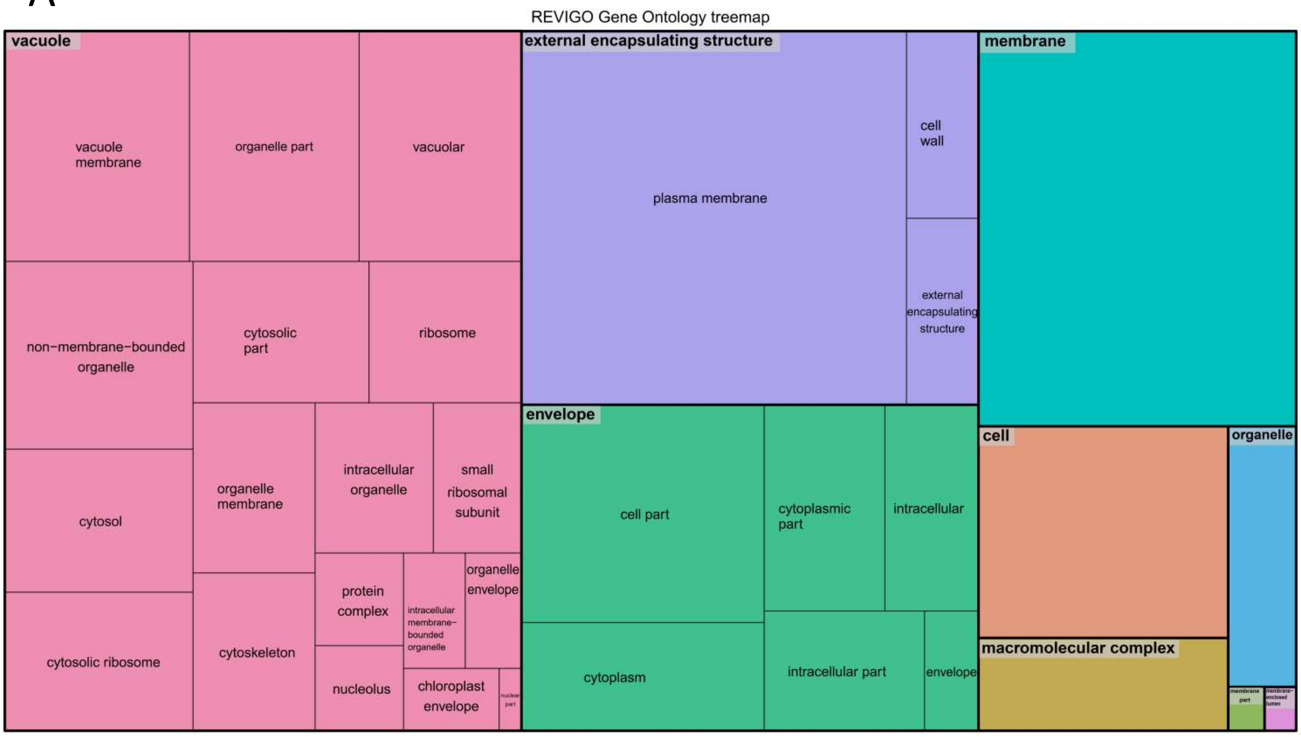

B

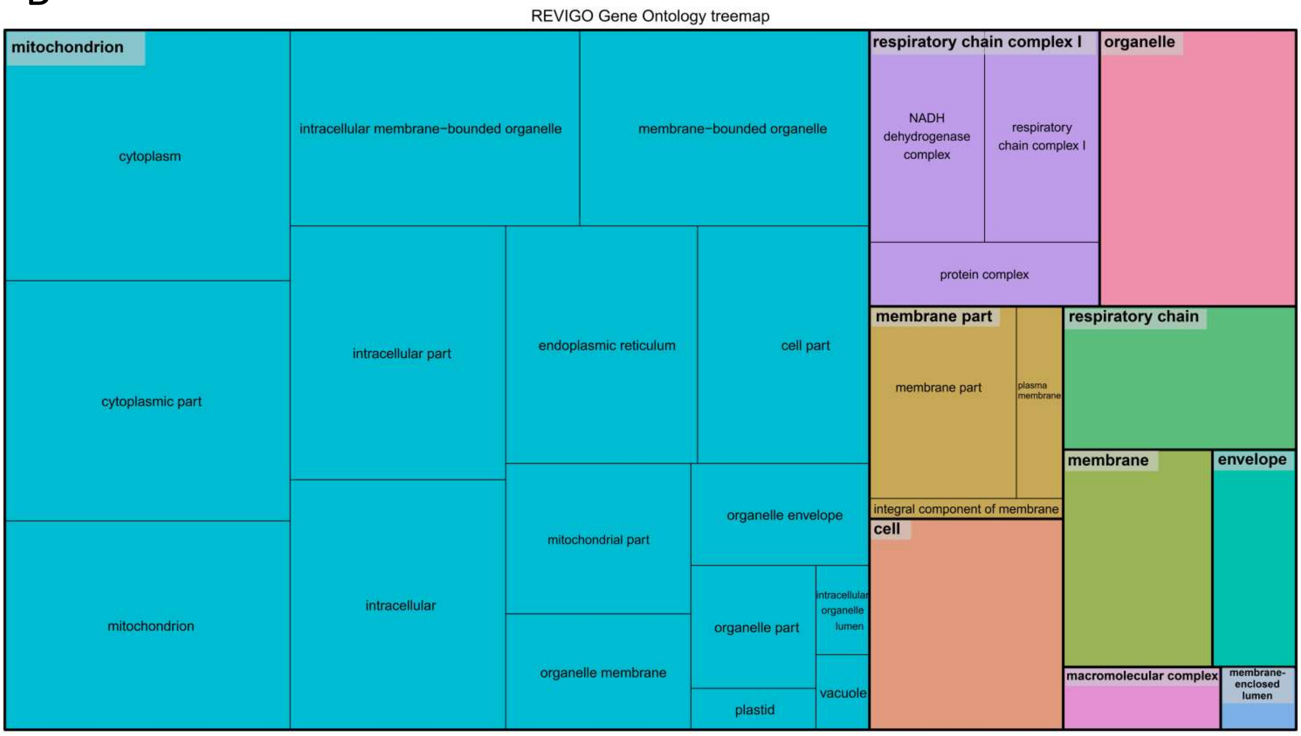

Fig. S2. REVIGO treemaps showing cellular component of sodium deoxycholate (SDC)-solubilized upper phase (UP) and microsomal (MF) fractions of Norway spruce developing xylem. A) Protein identifications specific for UP, and for B) MF.

A

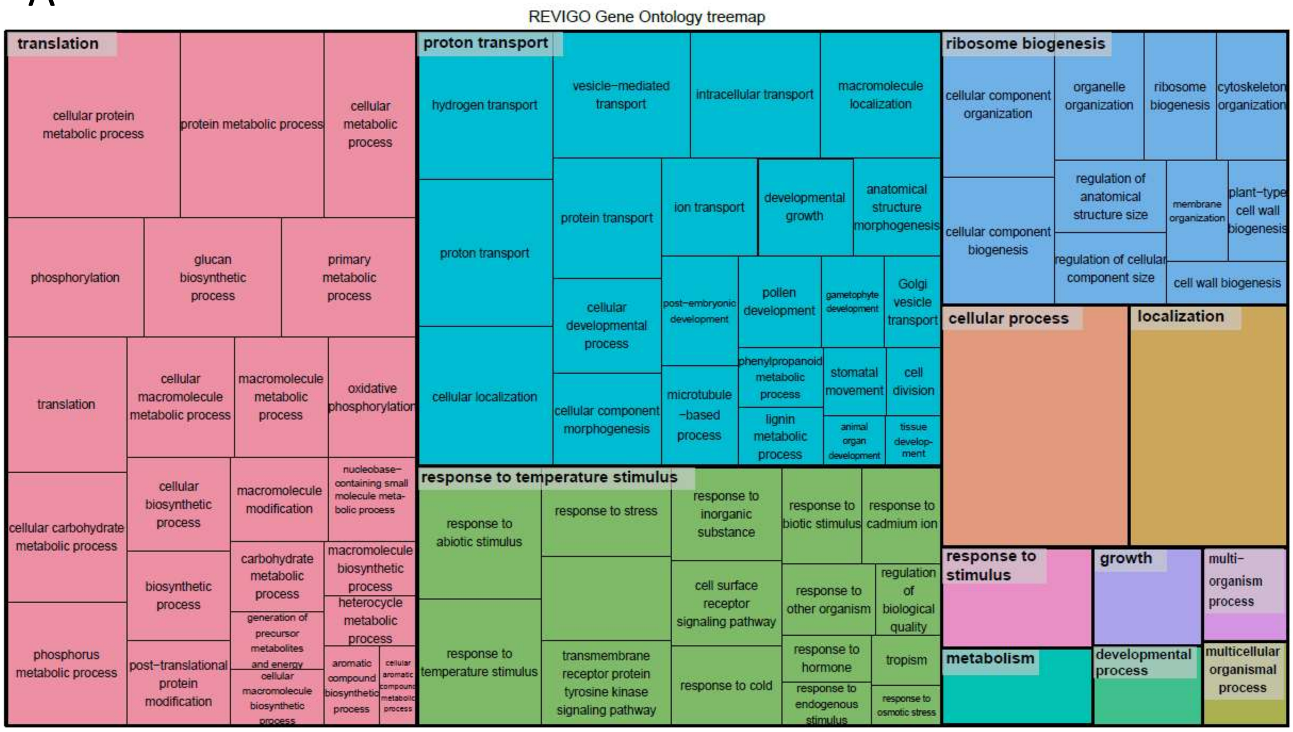
REVIGO Gene Ontology treemap
translation
cellular protein metabolic process
protein metabolic process
cellular metabolic process
phosphorylation
glucan biosynthetic process
primary metabolic process
translation
cellular macromolecule metabolic process
macromolecule metabolic process
oxidative phosphorylation
cellular carbohydrate metabolic process
cellular biosynthetic process
macromolecule modification
nucleobase-containing small molecule metabolic process
carbohydrate metabolic process
macromolecule biosynthetic process
heterocycle metabolic process
biosynthetic process
generation of precursor metabolites and energy
phosphorus metabolic process
post-translational protein modification
cellular macromolecule biosynthetic process
aromatic compound biosynthetic process
proton transport
hydrogen transport
vesicle-mediated transport
intracellular transport
macromolecule localization
protein transport
ion transport
developmental growth
anatomical structure morphogenesis
proton transport
cellular developmental process
post-embryonic development
pollen development
gametophyte development
Golgi vesicle transport
phenylpropanoid metabolic process
stomatal movement
cell division
cellular localization
cellular component morphogenesis
microtubule-based process
lignin metabolic process
animal organ development
tissue development
ribosome biogenesis
cellular component organization
organelle organization
ribosome biogenesis
cytoskeleton organization
regulation of anatomical structure size
membrane organization
plant-type cell wall biogenesis
cellular component biogenesis
regulation of cellular component size
cell wall biogenesis
cellular process
localization
response to temperature stimulus
response to abiotic stimulus
response to stress
response to inorganic substance
response to biotic stimulus
response to cadmium ion
cell surface receptor signaling pathway
response to other organism
regulation of biological quality
response to temperature stimulus
transmembrane receptor protein tyrosine kinase signaling pathway
response to cold
response to hormone
tropism
response to endogenous stimulus
response to osmotic stress
response to stimulus
growth
multi-organism process
metabolism
developmental process
multicellular organismal process

B

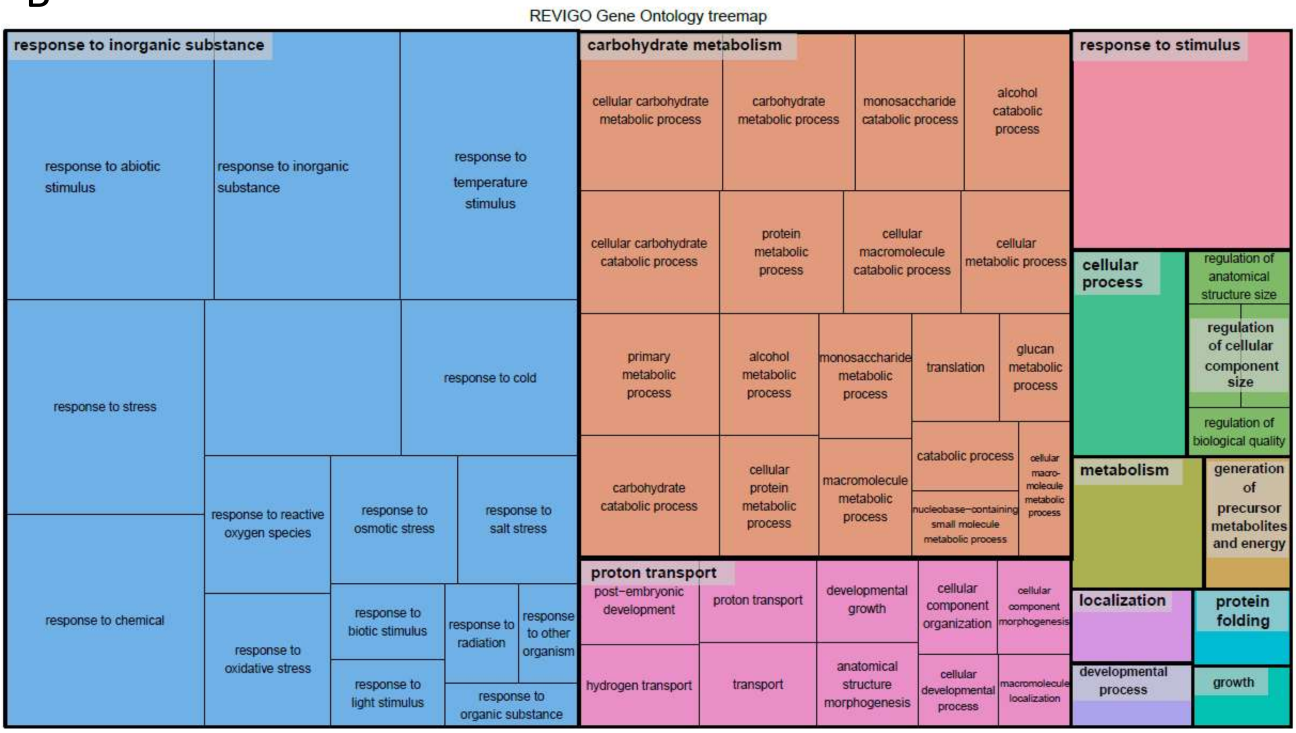
REVIGO Gene Ontology treemap
response to inorganic substance
response to abiotic stimulus
response to inorganic substance
response to temperature stimulus
response to stress
response to cold
response to chemical
response to reactive oxygen species
response to osmotic stress
response to salt stress
response to oxidative stress
response to biotic stimulus
response to radiation
response to other organism
response to light stimulus
response to organic substance
carbohydrate metabolism
cellular carbohydrate metabolic process
carbohydrate metabolic process
monosaccharide catabolic process
alcohol catabolic process
cellular carbohydrate catabolic process
protein metabolic process
cellular macromolecule catabolic process
cellular metabolic process
primary metabolic process
alcohol metabolic process
monosaccharide metabolic process
translation
glucan metabolic process
catabolic process
cellular macromolecule metabolic process
carbohydrate catabolic process
cellular protein metabolic process
macromolecule metabolic process
nucleobase-containing small molecule metabolic process
proton transport
post-embryonic development
proton transport
developmental growth
cellular component organization
cellular component morphogenesis
hydrogen transport
transport
anatomical structure morphogenesis
cellular developmental process
macromolecule localization
response to stimulus
cellular process
regulation of anatomical structure size
regulation of cellular component size
regulation of biological quality
metabolism
generation of precursor metabolites and energy
localization
protein folding
developmental process
growth

C

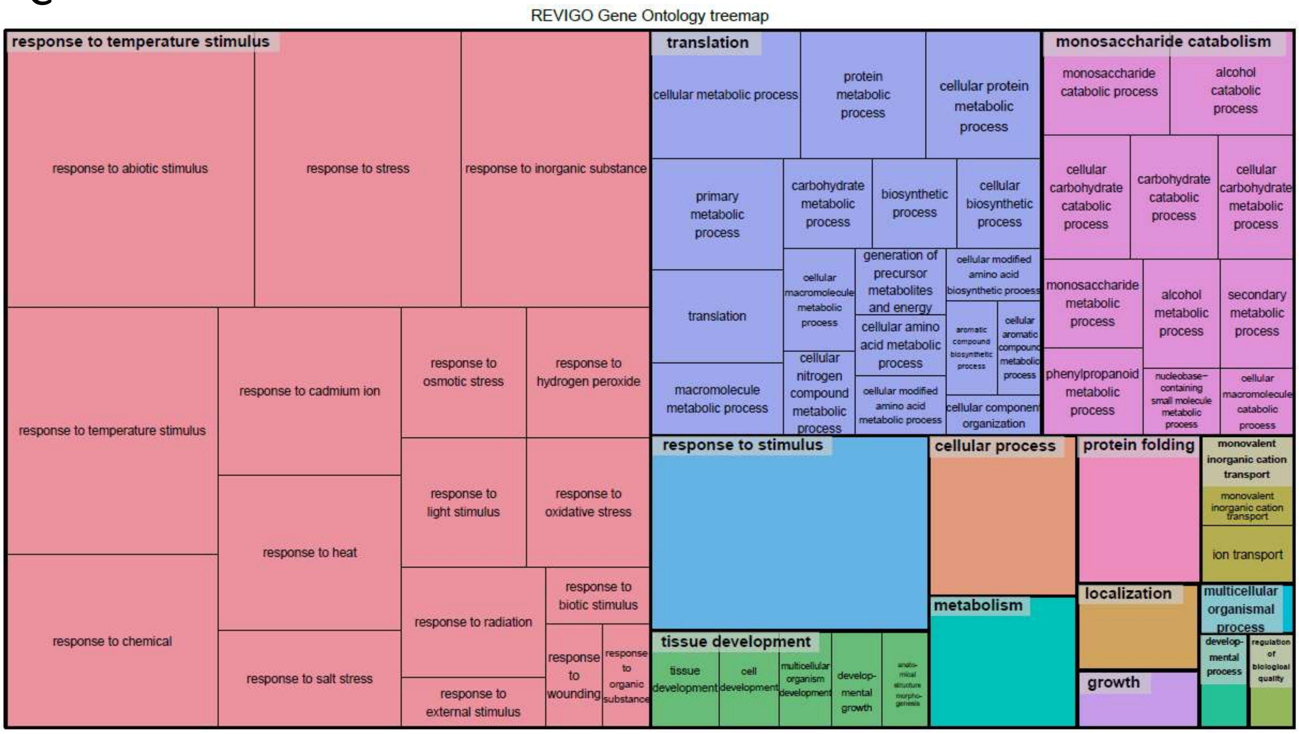

Fig. S3. REVIGO treemaps showing biological function of sodium deoxycholate (SDC)- and sodium dodecyl sulphate (SDS) -solubilized upper phase (UP) fractions of A) Norway spruce developing xylem, B) developing phloem, and C) lignin-forming tissue-cultured cells.

Hunting monolignol transporter(s): membrane proteomics and biochemical transport assays with membrane vesicles of Norway spruce

Enni Väisänen, Junko Takahashi, Ogonna Obudulu, Joakim Bygdell, Pirkko Karhunen, Olga Blokhina, Teresa Laitinen, Teemu H. Teeri, Gunnar Wingsle, Kurt V. Fagerstedt and Anna Kärkönen

Supplementary Protocol S1. ^{14}C-coniferin synthesis

Chemical formulas for the intermediates for the ^{14}C-coniferin synthesis (noted by a number in brackets) can be seen in the end of the Supplemental Protocol S1. ^{14}C-coniferin was synthesized as follows: vanillin was etherified with tetra-O-acetyl-α-D-glucopyranosylbromide according to Terashima et al. (1995) yielding compound (**1**). Next, the Knoevanagel reaction was performed as in Beejmohun et al. (2004, 2006). Compound (**1**) (400 mg, 0.83 mmol), ^{14}C-labeled malonic acid (9.6 mg, 0.091 mmol, activity 5 mCi) in pyridine (3 ml) and piperidine (3 drops) were mixed in a round bottomed flask. The reaction mixture was heated in an oil bath (+60-62 °C) for 20 min. After that the non-labelled malonic acid (120 mg, 1.15 mmol) was added, and the temperature kept at +90-95 °C for 2.5 h. The reaction was monitored with TLC. The reaction mixture was cooled and poured to ice water and acidified with HCl. The light brownish precipitate (compound **2**) was filtered in suction and washed several times with ice water. Yield of (**2**) was 313 mg (72 %). The product was pure on TLC, the unlabeled acid as a reference compound.

For esterification (as in Klán et al. 1992, Moore et al. 1979, Beejmohun et al. 2004, 2006), the compound (**2**) (313 mg, 0.60 mmol) was dissolved in 15 ml of acetone:water (95:5). $KHCO_3$ (120 mg, 1.2 mmol) was added followed by iodoethane (180 mg, 1.2 mmol) and tetrabutyl ammonium iodide (40 mg) after ca. 15 min. The reaction mixture was heated at +60 °C for 5 h. TLC analysis revealed some unreacted acid (**2**), and thus additional $KHCO_3$, iodoethane and tetrabutyl ammonium iodide were added and left for 20 h at room temperature. The addition of the reagents was repeated as there was unreacted acid (**2**) left, and the mixture heated at +60 °C for 2 h. After that the reaction was finished, and the ester (**3**) isolated. Acetone was evaporated in a stream of nitrogen, and the product was extracted with ethyl acetate. The organic phase was washed twice with a freshly prepared aqueous solution of $NaHCO_3$ (10%), water, an aqueous NaCl solution (25%), and dried with Na_2SO_4. Ethyl acetate was evaporated in a nitrogen stream. The yield of (**3**) was 297 mg (92 %), and the product was pure on TLC, and it was used without any further purification.

The reduction with diisobutylaluminium hydride (DIBAL-H) was performed according Terashima et al. (1995). In a three-necked round-bottomed flask, ester (**3**) (297 mg, 0.55 mmol) was dissolved in toluene (25 ml, distilled over sodium) under argon atmosphere. The reaction mixture was cooled in an ice bath, and 5.5 ml of 1.5 M diisobutylaluminium hydride in toluene was added with a syringe through a septum during 30 min. The mixture was kept cold and stirred for 1.5 h. Ethanol (5 ml) was carefully added, and stirring at room temperature was continued for ca. 45 min. Solvents were evaporated in a nitrogen stream, and the flask was kept in a warm water bath (ca. +60 °C). Water (20 ml) was added, and the product extracted into the water phase by warming the flask at ca. +80 °C, and filtered. The extraction was repeated twice. Concentration of the water filtrate yielded a yellowish raw product which was recrystallized from water producing a white powder. ^{14}C-Coniferin (**4**) was detected to be pure on RP-18 TLC (acetonitrile:water, 1:1). The yield of ^{14}C-coniferin was 121 mg (64 %), with some product in the mother liquor. Specific activity was determined by scintillation counting as 104 kBq µmol^{-1}.

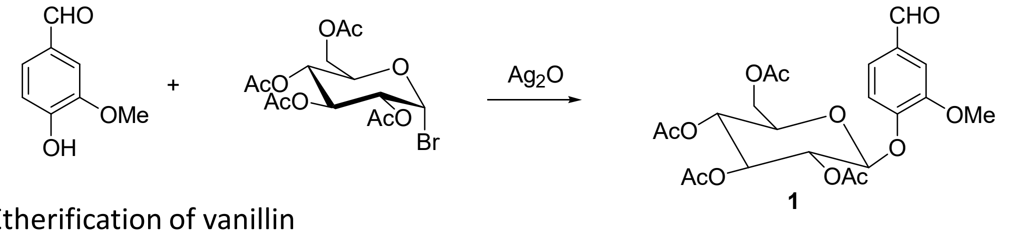

Etherification of vanillin

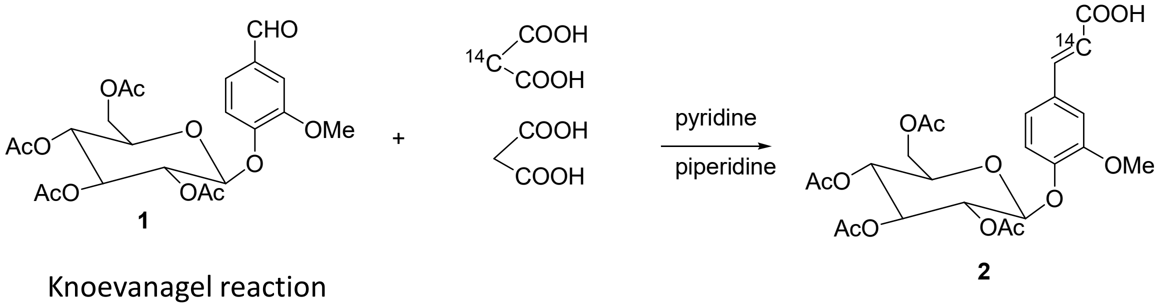

Knoevanagel reaction

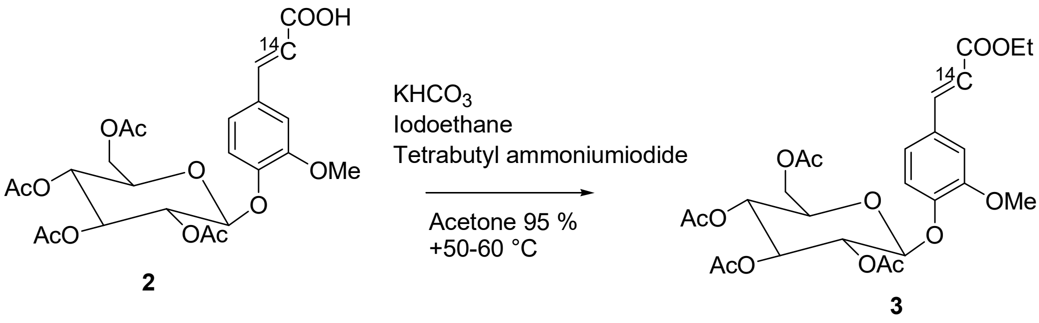

Esterification

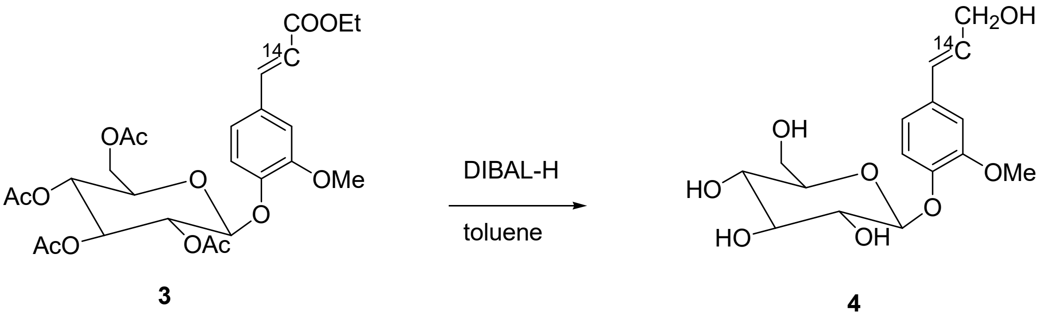

Reduction with DIPAL-H

References:

Terashima N, Ralph SA, Landucci LL. 1995. New facile syntheses of monolignol glucosides; *p*-glucocoumaryl alcohol, coniferin and syringin. Holzforschung **50,** 151-155.

Beejmohun V, Grand E, Mesnard F, Fliniaux M-A, Kovensky J. 2004. First synthesis of (1,2-$^{13}C_2$)-monolignol glucosides. Tetrahedron Letters **45,** 8745-8747.

Beejmohun V, Grand E, Lesur D, Mesnard F, Fliniaux M-A, Kovensky J. 2006. Synthesis and purification of (1,2-$^{13}C_2$)coniferin. Journal of Labelled Compounds and Radiopharmaceuticals. **49,** 463-470.

Klán P, Beňovský P. 1992. Phase-transfer catalyzed synthesis of 2-propenyl esters of carboxylic acids. Monatshefte für Chemie **123**, 469-472.

Moore GG, Foglia TA, McGrahan TJ. 1979. Preparation of hindered esters by the alkylation of carboxylate salts with simple alkyl halides. Journal of Organic Chemistry **44,** 2425-2429.
